# Phase-Based Dynamic Prediction of Delayed Cerebral Ischemia After Aneurysmal Subarachnoid Hemorrhage: Comparison of a Large Language Model with Intensive Care Specialists

**DOI:** 10.3390/medicina62091680

**Published:** 2026-09-02

**Authors:** Mustafa Ay, Dilara Tüfek Öztan, Şule Asri, Arzu Karaveli, Nazife Öztürk, Ahmet Şükrü Alparslan, Serap Avcı Ay

**Affiliations:** 1 Department of Intensive Care, Antalya Training and Research Hospital, 07100 Antalya, Türkiye; tufekdilara@gmail.com (D.T.Ö.); drsuleasri@gmail.com (Ş.A.); 2 Department of Anesthesiology and Reanimation, Antalya Training and Research Hospital, 07100 Antalya, Türkiye; 3 Research and Development Department, Antalya Training and Research Hospital, 07100 Antalya, Türkiye; 4 Department of Radiology, Antalya Training and Research Hospital, 07100 Antalya, Türkiye; 5 Department of Anesthesiology and Reanimation, Kemer State Hospital, 07980 Antalya, Türkiye

**Keywords:** delayed cerebral ischemia, aneurysmal subarachnoid hemorrhage, large language model, clinical decision support, risk prediction, intensive care

## Abstract

*Background and Objectives:* Delayed cerebral ischemia (DCI) is a major determinant of poor outcome after aneurysmal subarachnoid hemorrhage (aSAH), yet early risk prediction remains difficult, particularly in sedated or ventilated intensive care patients. We evaluated whether a large language model (LLM) could predict DCI from phase-based dynamic clinical data, compared with intensive care specialists. *Materials and Methods:* In this single-center, retrospective study, 216 consecutive patients with aSAH were assessed at three predefined phases of accumulating clinical data (day 1; days 1 + 3; days 1 + 3 + 5). For each patient–phase, an LLM (ChatGPT, GPT-5.5 Thinking) and two blinded intensive care specialists predicted DCI risk using only the data available up to that time point. DCI was adjudicated by a blinded three-member panel. Discrimination was assessed by the area under the receiver operating characteristic curve (AUC), with non-inferiority defined a priori as Δ = 0.10. Calibration, decision-curve analysis, and reproducibility were also assessed. *Results:* Of 216 patients, 60 (27.8%) were DCI-positive and 15 were indeterminate; the primary sample comprised 201 patients. In the prespecified primary analysis, the LLM met the non-inferiority criterion relative to both specialists across all three phases (LLM AUC 0.703–0.747; specialists 0.712–0.764); however, in an equal-granularity sensitivity analysis, non-inferiority remained supported only in Phases 2 and 3 and was not demonstrated in Phase 1. Discrimination increased numerically as data accumulated (Phase 1 vs. 3, *p* = 0.072), an increase that was attenuated in a landmark-restricted analysis accounting for DCI-onset timing. The LLM showed the lowest false-reassurance rate (3.3–11.7%), reflecting a more cautious threshold rather than better discrimination. Confidence did not reliably indicate accuracy (~25% of high-confidence predictions were wrong); outputs were highly reproducible (Fleiss κ 0.887; intraclass correlation coefficient (ICC) 0.968). *Conclusions:* An LLM achieved DCI discrimination that was non-inferior to—but not better than—that of experienced specialists; its unreliable confidence scores support clinician-supervised rather than autonomous use.

## 1. Introduction

Aneurysmal subarachnoid hemorrhage (aSAH) is a life-threatening condition characterized by high mortality and persistent neurological sequelae in critical care practice [1,2,3]. Delayed cerebral ischemia (DCI) is a critical complication in these patients that typically develops between days 3 and 14 after the hemorrhage and is regarded as one of the principal determinants of mortality and poor functional outcome [4].

The development of DCI cannot be explained by macrovascular vasospasm alone; microcirculatory dysfunction, the inflammatory response, cortical spreading depolarizations, early brain injury, and metabolic derangements all contribute to this process [5]. This multifactorial pathophysiology hampers accurate early prediction of DCI risk and complicates clinical decision-making, particularly in intensive care patients whose neurological examination is constrained by sedation, mechanical ventilation, or poor neurological status.

Risk assessment in patients with aSAH relies largely on baseline clinical and radiological scoring systems. Tools such as the World Federation of Neurosurgical Societies (WFNS) grade, the modified Fisher scale, and the Subarachnoid Hemorrhage Early Brain Edema Score (SEBES) are useful for assessing disease severity, hemorrhage burden, and early brain edema [6,7,8]. However, these static scores may not fully reflect the inflammatory, metabolic, and hemodynamic changes that develop over the course of intensive care. DCI risk should therefore be evaluated not only on the basis of admission data but also using clinical and laboratory variables that change over time.

Artificial intelligence- and machine learning-based approaches are increasingly used in clinical risk prediction and decision-support systems [9,10,11]. Large language model (LLM) technologies, by virtue of their potential to process clinical data—whether structured or in natural-language form—in a context-aware manner, constitute a novel methodological avenue in clinical prediction research [12,13,14]. Although LLMs have been shown to encode a broad base of medical knowledge, important limitations regarding factual accuracy, bias, and safety have also been reported [15]. However, their performance and reliability for phase-based risk prediction in the intensive care setting—particularly when patient data change over time—remain insufficiently characterized.

Much of the existing AI literature focuses on overall performance metrics, whereas the direction of error, calibration, and model reliability—all critical for clinical decision-making—remain insufficiently examined. High-confidence errors are of particular concern, as they can create a misleading sense of certainty in clinical practice and lead to delayed or inadequate intervention [16]. In LLM studies, transparent reporting of details such as model version, prompt structure, input data, output format, the use of independent sessions, and error analysis is essential for reproducibility; current LLM-specific reporting guidelines emphasize that these elements should be prespecified [17,18].

The aim of this study was to evaluate the performance of an LLM (ChatGPT, GPT-5.5 Thinking) in predicting the risk of DCI using phase-based dynamic clinical data in patients managed in the intensive care unit (ICU) for aSAH, to compare this performance with that of intensive care specialists, and to assess the model’s reliability in terms of confidence levels and error patterns. In addition, to provide a structured assessment of clinical reasoning, the contribution of five clinical evaluation domains—neurological severity, radiological burden, inflammatory response, hemodynamic instability, and metabolic derangement—was examined separately for each domain, and the reasoning patterns of the model and the clinicians were compared using domain-specific agreement analyses. Throughout this study, the LLM was evaluated as an investigational risk-assessment approach intended for clinician-supervised decision support rather than as an autonomous diagnostic or bedside decision-making tool.

## 2. Materials and Methods

### 2.1. Study Design and Setting

This study was conducted as a single-center, observational, phase-based prediction-performance study in which patients managed for aSAH in a tertiary intensive care unit (ICU) were retrospectively evaluated. It was carried out between January 2020 and January 2026.

The study was conducted in accordance with the Declaration of Helsinki and was approved by the Ethics Committee of Antalya Training and Research Hospital on 7 May 2026 (decision no. 9/2). Owing to the retrospective design, the requirement for individual informed consent was waived by the ethics committee.

The study was designed and reported in accordance with the STROBE statement for observational clinical research and the TRIPOD-LLM guideline for reporting prediction studies based on large language models, while also taking the TRIPOD + AI recommendations into account where relevant. The data inputs, the model and expert evaluation process, the output format, the reference standard, and the performance analyses were all prespecified [17,18].

### 2.2. Patient Selection

Consecutive patients aged 18 years or older with a diagnosis of aSAH confirmed by imaging (CT angiography [CTA] or digital subtraction angiography [DSA]) were included. For each patient, only the first intensive care admission for the index hemorrhage was evaluated.

Patients with repeat admissions beyond the index admission, as well as those lacking sufficient clinical follow-up or appropriate follow-up imaging for DCI assessment, were excluded.

Because the study was based on a phase-based prediction design, each patient had to be evaluable across all three assessment phases. Accordingly, patients who died before day 5 or were transferred to another center—and for whom Phase 3 data could therefore not be generated—were excluded in a prespecified manner; in these cases, the day-5 clinical data required for Phase 3 were unavailable, so the three-phase comparison framework could not be completed, and their inclusion would have required artificial completion of the clinical dataset. The analyzed cohort therefore represents patients who passed the day-5 landmark and remained under follow-up.

The non-inferiority margin of Δ = 0.10 AUC was prespecified before comparative analyses. There is no universally established AUC-based non-inferiority margin for AI-versus-clinician studies; methodological guidance recommends that the margin be defined a priori according to the largest reduction in performance considered clinically and scientifically acceptable for the intended use [19]. Importantly, an absolute AUC margin of 0.10 has precedent in published diagnostic-performance studies, including an automated ASPECTS-scoring study in acute ischemic stroke in which algorithmic methods were compared with neuroradiologists using a 0.10 AUC non-inferiority margin [20]. In the present study, the LLM was evaluated as an investigational risk-stratification aid intended to complement, rather than replace, clinician assessment; its output was not intended to trigger autonomous diagnosis or treatment. Accordingly, Δ = 0.10 was prespecified as the maximum acceptable reduction in discrimination within this intended clinician-supervised decision-support context. We acknowledge that the choice of a non-inferiority margin is inherently context-dependent and that more stringent margins may be appropriate for other clinical applications.

### 2.3. Phase-Based Clinical Data Structure

The clinical course of the patients was examined across three phases in order to assess time-dependent changes: Phase 1, day 1 data; Phase 2, day 1 and day 3 data; and Phase 3, day 1, day 3, and day 5 data.

These phases were selected on a clinical and pathophysiological basis to represent the early brain injury period (the first 72 h) and the DCI development window. Within each phase, only the clinical, laboratory, radiological, and treatment data available up to the corresponding time point were included. Information from later time points and outcome data were withheld from both the LLM and the clinician assessors. Each patient–phase combination was treated as a separate prediction task. The exact calendar day of DCI onset was not initially captured as a discrete variable in the primary dataset; DCI status was instead determined for the days 3–14 window as a whole by the adjudication panel (see Primary outcome and adjudication).

To preserve information symmetry between the two assessor groups (the LLM and the human clinicians), the formal study-phase label (Phase 1, Phase 2, or Phase 3) was not provided to either assessor. However, the day-specific headings within the presented dataset (e.g., Day 1 and Day 3 data) remained visible. Thus, both assessors could identify the temporal position of the assessment from the study days represented in the available data, but they were not explicitly informed of the corresponding formal phase designation. This design was intended to reduce assessor-specific contextual cues, so that observed performance differences would more plausibly reflect differences in clinical data interpretation rather than phase-related context.

### 2.4. Clinical Variables

**Baseline (static) variables.** Age, sex, comorbidities, admission and procedure times, WFNS grade, modified Fisher scale, SEBES score, intraventricular hemorrhage, hydrocephalus, intraparenchymal hematoma, midline shift, pupillary status, aneurysm location, anterior versus posterior circulation, and number of aneurysms were recorded. These variables were selected because they reflect baseline neurological severity, hemorrhage burden, and structural radiological features.

**Early treatment and initial management variables.** Time to aneurysm securing, treatment modality (coiling or clipping), nimodipine use, external ventricular drainage (EVD), and antithrombotic medication use were recorded. These variables reflect early clinical management and treatment approach and were provided for assessment as phase-based input variables potentially available before DCI onset.

**Phase-based dynamic clinical and laboratory variables.** Glasgow Coma Scale (GCS), sedation, mechanical ventilation, vasopressor use, infection status, leukocyte, neutrophil, and lymphocyte counts (×10^3^/mm^3^), neutrophil-to-lymphocyte ratio (NLR), C-reactive protein (CRP, mg/L), creatinine (mg/dL), sodium (mmol/L), glucose (mg/dL), and lactate (mmol/L) were recorded. These variables were grouped under the five clinical evaluation domains of neurological severity, radiological burden, inflammatory response, hemodynamic instability, and metabolic derangement. In each phase, the most recent values available up to the corresponding time point were included in the analysis.

### 2.5. Radiological Scoring and Imaging Assessment

Baseline CT and angiographic imaging findings were recorded from patient charts and radiology reports. When possible, the modified Fisher scale and the SEBES score were based on the initial admission CT or on the documented radiological assessment.

### 2.6. Primary Outcome and Adjudication

The primary outcome was an adjudicated composite DCI outcome, defined as clinical deterioration attributable to DCI and/or a new cerebral infarction attributable to DCI. Clinical DCI was defined as neurological deterioration occurring between days 3 and 14 after the hemorrhage that was not attributable to alternative causes. Neurological deterioration was defined as a decrease of at least 2 points on the GCS or a new focal neurological deficit lasting at least 1 h [4].

During adjudication, alternative causes such as rebleeding, hydrocephalus, seizures, metabolic derangements, infection/sepsis, and hemodynamic instability were systematically excluded. In patients in whom neurological assessment was limited, imaging-based DCI was defined as the presence of a new cerebral infarction on follow-up imaging that was not attributable to the aneurysm-securing procedure or another identifiable cause. The DCI diagnostic criteria were applied based on the 2010 consensus definition of Vergouwen et al. [4].

The primary outcome was adjudicated by a three-member multidisciplinary panel, blinded to the model and clinician predictions, using a structured checklist (Supplementary File S3). The panel comprised an anesthesiology and reanimation specialist; an intensive care specialist with a background in anesthesiology and reanimation and subspecialty certification in intensive care; and a radiologist specialized in neuroradiology. Each panel member assessed every case independently; in the event of unanimous agreement, the result was accepted directly, whereas disagreements were resolved by majority decision (2 of 3), and a single final outcome column was reported. The panel members who performed outcome adjudication were independent of the two clinicians who provided the risk predictions, and no individual served in both roles. Individual pre-consensus adjudication votes were not retained; therefore, inter-rater agreement before consensus could not be quantified retrospectively.

For the landmark-restricted sensitivity analysis described below, the adjudication panel subsequently reviewed the source clinical records of the 60 DCI-positive patients and documented the day, relative to hemorrhage onset, on which the qualifying clinical or imaging evidence for DCI was first recorded. This day was determined from the same documentation used for the original adjudication (nursing GCS charts, clinical notes, and imaging timestamps) rather than from recollection, and was successfully determined for all 60 DCI-positive patients.

Following adjudication, cases were classified as DCI-positive, DCI-negative, or indeterminate. The indeterminate category was used to denote cases in which neurological assessment was limited, clinical and imaging findings were conflicting, or alternative causes could not be fully excluded. The primary analysis included only cases that could be definitively classified, and indeterminate cases were addressed separately in sensitivity analyses.

### 2.7. Secondary Clinical Outcomes

Secondary outcomes were ICU mortality, ICU length of stay, the presence of vasospasm, and the need for decompressive surgery. Vasospasm was recorded as cerebral arterial narrowing detected on clinical grounds and/or by methods such as CTA, DSA, or transcranial Doppler. Vasospasm alone was not regarded as DCI; DCI was assessed according to the primary outcome definition.

### 2.8. Large Language Model (ChatGPT) Evaluation

The LLM used in this study was ChatGPT (OpenAI; interface-displayed model label: “GPT-5.5 Thinking”), accessed through the web interface (chatgpt.com) under a pre-existing personal ChatGPT Plus subscription rather than an account created specifically for this study. Memory, Reference Chat History, and Custom Instructions were disabled throughout all evaluations, and each patient–phase assessment was conducted in a new, blank chat session. No backend deployment identifier or other stable version identifier was exposed to the investigators through the commercial web interface; therefore, “GPT-5.5 Thinking” refers to the model label displayed in the user interface at the time of evaluation. All evaluations were performed in Antalya, Türkiye, using the same browser, account, and interface environment (a new, independent chat conversation was opened for each patient–phase evaluation; see below). The pilot phase was conducted on 16 May 2026, and the main batch of evaluations was carried out between 16 and 18 May 2026, after completion of the pilot, using the same interface-displayed model label. Model evaluations were performed with a fixed, prespecified prompt applied to all patients (Supplementary File S2).

A new, independent chat session was opened for each patient and each phase; to prevent responses from earlier patients or phases from influencing subsequent evaluations, no sequential patient evaluations were performed within the same session, and each session was initiated as a blank conversation. Throughout all evaluations, the Memory, Reference Chat History, and Custom Instructions features were kept disabled, and the same browser, user account, and interface-displayed model label were maintained for the duration of the study. Details on the documentation of these settings and of the evaluation environment are provided in the Section 2.10.

The model was provided only with the patient data available up to the corresponding phase. Outcome data—the DCI adjudication result, mortality, vasospasm outcome, and information from subsequent days—were not included in the prompt. Because the web interface was used, model parameters, most notably temperature, could not be controlled at the user level, and evaluations were conducted under the interface’s default conditions at that time. This approach reflects the conditions under which clinicians access consumer-grade AI tools in routine practice and prioritizes ecological validity.

The model was asked to produce a structured output for each patient–phase evaluation: (i) the DCI risk category (low/moderate/high); (ii) the estimated DCI probability (a single integer percentage, 0–100); (iii) the confidence level in this risk estimate (a single integer percentage, 0–100); and (iv) the contribution of each of the five clinical evaluation domains (neurological severity, radiological burden, inflammatory response, hemodynamic instability, and metabolic derangement) to the DCI risk prediction, with one of the options “supports,” “no notable contribution,” or “inconsistent with the prediction” requested for each domain.

To avoid reducing clinical reasoning to a single dominant factor, a comprehensive rating approach was adopted instead of the conventional forced single-choice format: an independent three-category rating was applied to each of the five clinical domains. To avoid the ambiguity inherent in “positive/negative” terminology, coherence-oriented wording (“supports”/“inconsistent with the prediction”) was used. This structure was intended to reduce the response bias introduced by conventional “multifactorial” or “indeterminate” escape-hatch categories and to enable domain-specific agreement analyses.

The model was instructed not to generate treatment recommendations, references, disclaimers, or any text beyond the requested format. Responses that fell outside the structured format were reviewed according to predefined data-cleaning rules and excluded from the analysis where necessary.

### 2.9. Pilot Study and Prompt Locking

Before the main data collection, a pilot study comprising 9 prompts (3 patients × 3 phases) was conducted, with three patients selected to span the clinical severity spectrum (low severity, moderate-to-high severity, and sedated). The aims of the pilot were (a) to test whether the prompt format allowed the model to produce consistent, structured output and (b) to verify the response-recording workflow (PDF, screen recording, and raw record) in practice. All 9 pilot responses were obtained in the required four-item structured format, in accordance with the predefined rules, and no disclaimers, references, or off-format text were produced. Because no problems with the format or the recording workflow were identified, the prompt was locked at version 2.0 and was not modified throughout the main batch. As no changes were made to the prompt or the recording workflow during the pilot, these nine evaluations were retained as the main-batch outputs for the corresponding patient–phase records.

**Management of non-standard responses.** The following rules for non-standard LLM outputs were defined a priori, before data collection: (i) when one or more structured fields were missing, completion was requested once within the same chat session; (ii) off-format responses that nonetheless contained substantive content were assigned to the highest category according to a predefined coding rule; (iii) off-format responses, treatment recommendations, or refusals to provide an assessment were re-queried once in a new, blank chat session, and responses that remained unsuitable on the second attempt were coded as “not evaluable.” These rules served as a prespecified audit framework during data collection. In the main evaluation (648 patient–phases), no non-standard response requiring exclusion occurred, and all evaluations were obtained in structured format.

**Prompt language and rationale.** The prompts were prepared in Turkish. This choice was based on two considerations: (1) preserving information symmetry between the model and the clinicians, given that the two intensive care specialists in the study performed their clinical assessments in Turkish; and (2) reflecting real-world usage conditions in the Turkish clinical setting (ecological validity). The Turkish originals of the prompts and their English translations are provided in Supplementary File S2. The raw pilot outputs and audit records (time-stamped PDF records, ChatGPT chat URLs, screenshots, and screen recordings) contain patient-level data and are therefore not deposited as a public additional file; they are retained and are available to the editors and reviewers on reasonable request, subject to privacy and ethical restrictions.

### 2.10. Data Recording and Audit Protocol

To ensure reproducibility and auditability, a prespecified audit-trail protocol was applied. For every patient–phase evaluation, the following were maintained: (i) a PDF record of all prompt and response content after each chat (content evidence; the PDF carries an automatic date stamp and the ChatGPT chat URL in the top corner); (ii) archiving of each chat in the ChatGPT archive (providing access to the original conversation and a server-side, independent timestamp); and (iii) copying of the full response text into a raw-record document together with the record number, the patient–phase label, the date and time, and the model details (a searchable text record). In addition, for environmental and process verification, a screenshot showing the model name and the system clock was captured at the start of each evaluation session, along with a video recording covering all phases of one or two patients (three in some sessions) from that session; the pilot phase was recorded on video in its entirety. Video and screenshots were obtained at the session level rather than for each individual query. The fixed study information (platform, account type, interface-displayed model label, settings, location, and prompt version) was defined once at the beginning of each raw-record file, and each record contains only the variable fields (record number, patient–phase, date and time, prompt, and response). Structured output fields (risk category, probability, confidence, and the five domain attributions) were manually transcribed by the corresponding investigator (M.A.) from the preserved response records into the study database. The original PDF, archived-chat, and raw-record materials were retained as an audit trail for the extracted values. An independent double-check of the transcribed values by a second investigator was not performed.

### 2.11. Intensive Care Specialist Assessment

The expert assessments were performed by two independent physicians with 23 and 24 years of clinical experience, including 14 and 16 years of intensive care experience, respectively. Both were long-standing members of the tertiary-center ICU team and were routinely involved in the management of patients with aSAH through regular daytime and on-call clinical duties. The center managed approximately 40 aSAH ICU admissions annually during the study period. The assessors worked blinded to the patients’ DCI outcome, the adjudication result, and the LLM output. The clinician assessors were independent of the specialists who performed the outcome adjudication, and no individual served in both roles.

For each patient, the specialists were provided only with the data available up to the corresponding phase. The phase assessments were performed independently, and no information from later time points was provided. In keeping with the information-symmetry principle applied to the model, the clinicians were likewise not informed of which phase each assessment belonged to.

To realistically reflect clinical decision-making practice and to minimize assessor fatigue (fatigue bias), the clinicians were asked to select one of four predefined DCI probability ranges (0–25%, 26–50%, 51–75%, 76–100%) rather than provide a precise percentage estimate. Confidence in each prediction was recorded in three categories (low, 0–33%; moderate, 34–66%; high, 67–100%); this categorization was adopted for pragmatic comparability, drawing on the clinical decision-making and cognitive-bias literature [21,22].

The clinicians marked the contribution of each of the same five clinical evaluation domains to the DCI risk prediction using one of the options “supports,” “no notable contribution,” or “inconsistent with the prediction.”

LLM outputs were recorded as continuous probability values on a 0–100% scale. In addition to the four-level probability-range estimate, the clinicians recorded an overall DCI risk category (low/moderate/high). For continuous analyses (AUC, calibration, Brier score, and decision curve analysis), the LLM’s raw numerical outputs were used, the clinician probability categories were represented by the prespecified values 12.5, 37.5, 62.5, and 87.5, and the clinician confidence categories were likewise converted to a continuous variable (low → 17, moderate → 50, high → 84). Risk-category agreement and false-reassurance analyses were based on each assessor’s native low/moderate/high risk-category output. Domain-agreement analyses used the original three-level domain-attribution responses. Only in the equal-granularity sensitivity analysis was the LLM’s continuous probability output collapsed into the same four probability bands used by the clinicians.

### 2.12. Reproducibility Substudy

To assess the between-run stability of the LLM outputs, 20 patients were selected from the study population by systematic sampling. The systematic sampling interval was calculated as *k* = *N*/*n* ≈ 11 (216 total patients/20 in the subgroup); beginning with the first patient on the list, every 11th patient was included in the subgroup. This method was intended to distribute the subgroup across the entire patient series and reduce the risk of clustering within a particular period or subgroup.

Each phase of the 20 selected patients was queried in two further independent, newly opened chat sessions in addition to the main batch run (a total of 20 patients × 3 phases × 3 runs = 180 evaluations; of these, 60 were part of the main batch and 120 were the additional runs of the reproducibility substudy). Between-run agreement was assessed using Fleiss κ for the categorical variables (risk category and the five clinical evaluation domains) and using the intraclass correlation coefficient [ICC(2,1)] together with the absolute between-run difference for the numerical variables (probability and confidence level). The main performance analysis was conducted on the single-run outputs, and the reproducibility substudy served to evaluate the stability of this approach.

### 2.13. Statistical Analysis

Because this was a retrospective study that included all eligible consecutive patients during the predefined study period, no a priori sample-size calculation was performed. Statistical precision was instead evaluated using 95% confidence intervals around the AUC estimates and the paired AUC differences; the achieved sample size (*n* = 201 in the primary analysis; 60 DCI events) is reported so that readers can judge the precision of the reported estimates.

To address the possibility that some Phase 2 and Phase 3 predictor variables were recorded after DCI onset had already begun, a landmark-restricted sensitivity analysis was performed using the reconstructed DCI onset day. For each phase, DCI-positive patients whose documented onset preceded or coincided with that phase’s data-accrual landmark (Day 3 for Phase 2; Day 5 for Phase 3) were excluded, while all corresponding DCI-negative patients were retained; AUCs, paired AUC differences, and non-inferiority margins were recomputed in these landmark-restricted cohorts. To separate the effect of case-mix change from that of outcome leakage, Phase 1, Phase 2, and Phase 3 discrimination were also recomputed within the single common cohort of patients without documented DCI by Day 5, allowing a paired, same-population comparison of discrimination across phases.

Because the LLM produced continuous probability estimates whereas the clinicians selected one of four predefined probability ranges, the two assessor groups differed in output resolution; the clinicians’ four-level responses generate substantially more tied values, which can affect rank-based discrimination measures. To quantify the influence of this asymmetry, an equal-granularity sensitivity analysis was performed: the continuous LLM probabilities were collapsed into the same four intervals used by the clinicians (0–25%, 26–50%, 51–75%, 76–100%), and phase-wise AUCs, paired AUC differences, and non-inferiority conclusions were recomputed at this common resolution.

As an exploratory benchmark analysis, discrimination of the LLM was compared with three established baseline aSAH severity measures: WFNS grade, modified Fisher scale, and SEBES. AUCs with 95% CIs were calculated for each score, and paired DeLong tests were used to compare each phase-specific LLM AUC with the corresponding baseline-score AUC in the same patients. These analyses were considered exploratory; nominal two-sided *p* values are reported without adjustment for multiplicity.

Statistical analyses were performed in Python 3 (version 3.12; SciPy, scikit-learn, statsmodels, and pandas packages), and descriptive analyses were additionally verified with IBM SPSS Statistics (version 27). Statistical significance was set at a two-sided *p* < 0.05. Risk-category agreement and probability-correlation analyses included all 216 patients because these assessor-to-assessor comparisons did not depend on the adjudicated DCI reference standard; the 15 indeterminate cases were excluded only from outcome-based primary performance analyses.

After the distribution of continuous variables was assessed with the Shapiro–Wilk test, normally distributed variables were presented as mean ± standard deviation and the remainder as median (interquartile range); categorical variables were presented as counts and percentages. For between-group comparisons, the independent-samples t test or the Mann–Whitney U test was used for continuous variables, and the chi-square test—or the Fisher exact test when expected cell counts were low—was used for categorical variables. Cases in which the admission GCS could not be assessed because of sedation were treated as missing in analyses of the actual scores, and sedation status was reported separately.

**Primary performance analysis.** Primary performance was evaluated against the adjudicated definitive DCI outcome (DCI-positive/DCI-negative) as the reference standard, and indeterminate cases were excluded from the primary analysis. Discrimination was quantified by the area under the receiver operating characteristic (ROC) curve (AUC), and AUC values were reported with 95% confidence intervals (CIs). AUCs and paired AUC comparisons were computed using the DeLong method [23]. Each evaluation phase (Phase 1, Phase 2, and Phase 3) was analyzed separately.

The comparison framework was prespecified as non-inferiority, with a clinically meaningful non-inferiority margin of Δ = 0.10 AUC. Non-inferiority was accepted when the lower bound of the 95% CI for the AUC difference between the LLM and the comparator remained above −0.10. The primary comparisons were defined as the separate comparison of the LLM with each of the two intensive care specialists, whereas the comparison with the average of the two specialists’ probability estimates (the expert average) was defined as a supporting analysis. The expert average was prespecified as a supporting statistical construct rather than as a representation of routine bedside decision-making. It was intended to reduce the influence of idiosyncratic differences between individual assessors by averaging their probability estimates. It does not represent an actual clinical consensus process, and the primary comparisons therefore remained the separate comparisons between the LLM and each clinician. The *p* values from the DeLong test correspond to two-sided superiority comparisons, whereas the non-inferiority decision was based on the confidence interval. Dynamic change across phases was assessed by comparing the AUCs of successive phases and by comparing the Phase 1 and Phase 3 AUCs with the paired DeLong test.

**Classification and calibration.** In a secondary, descriptive classification analysis, the continuous LLM probability output was dichotomized for each phase at the threshold that maximized the Youden index, and sensitivity, specificity, and positive and negative predictive values (PPV and NPV) were calculated. Model calibration was assessed for the LLM, which produces continuous probabilities, using the Brier score and calibration curves based on equal-frequency (quintile) bins [24]. In addition, calibration intercept and slope were estimated for each phase using logistic calibration models relating the observed DCI outcome to the logit of the LLM-predicted probability; ideal calibration corresponds to an intercept of 0 and a slope of 1. Confidence intervals for sensitivity, specificity, PPV, and NPV were calculated using the Wilson score method. Because the clinician probabilities were derived from the prespecified representative values of the structured ranges, clinician calibration was reported as exploratory. An exploratory net-benefit-based decision curve analysis (DCA) [25] was performed; in this analysis, “action” (treat) refers not to a specific treatment but to classifying a patient as high-risk for intensive DCI monitoring or further imaging.

**Reasoning structure and reliability.** Inter-rater agreement was assessed using weighted (quadratic) Cohen’s κ for the risk category and Spearman correlation for the probability estimates. To compare the structure of clinical reasoning, domain-specific (unweighted) Cohen’s κ was calculated between the LLM and the clinicians for each of the five clinical evaluation domains; because discrimination and calibration were evaluated separately within each phase, each patient contributed only one observation to each phase-specific analysis, but this pooled domain analysis combined up to three correlated observations per patient. Within-patient dependence in this pooled analysis was therefore addressed using patient-level clustered bootstrap resampling (2000 resamples), in which all phase-level observations belonging to a resampled patient were kept together to generate 95% confidence intervals; point estimates were nonetheless interpreted as exploratory. Reproducibility estimates were additionally recalculated separately for each phase as a sensitivity analysis. The relationship between confidence level and accuracy was examined, and error rates among high-confidence predictions (confidence ≥ 75) were additionally evaluated. Assigning “low” risk to patients who developed DCI (false reassurance) was operationally defined as a clinically critical type of error and was reported separately for each assessor.

**Reproducibility.** The between-run stability of the LLM outputs was assessed across three independent runs in the 20 patients selected by systematic sampling. Between-run agreement was evaluated using Fleiss κ for the categorical outputs (risk category and the five clinical domains) and using the intraclass correlation coefficient [ICC(2,1); two-way random effects, absolute agreement, single measures] together with the mean absolute between-run difference for the continuous outputs (probability and confidence). For the reproducibility coefficients, 95% confidence intervals were obtained using 2000 patient-level clustered bootstrap resamples, in which all phase-level observations and all three runs belonging to a resampled patient were retained together; percentile confidence intervals are reported.

**Missing data, indeterminate cases, and multiplicity.** Missing values were conveyed explicitly as blank/missing in the LLM and clinician prompts; neither the model nor the clinicians were asked to impute or make assumptions about missing values. Cases with an indeterminate adjudication result were excluded from the primary analysis, and a sensitivity analysis was performed comparing three scenarios in which these cases were (a) excluded from the analysis (primary), (b) all treated as DCI-positive, or (c) all treated as DCI-negative. The analytic cohort as a whole was conditional on the availability of day-5 data and therefore represents a day-5 landmark cohort (see Patient selection for details); this is distinct from the landmark-restricted sensitivity analysis based on reconstructed DCI onset described below. To address the risk of multiple comparisons, the primary hypothesis (non-inferiority of the LLM to each individual clinician) was prespecified, whereas the comparison against the expert average and the reasoning, reliability, and clinical-utility analyses were interpreted as supporting/exploratory.

## 3. Results

### 3.1. Patient Characteristics

**Figure 1:**
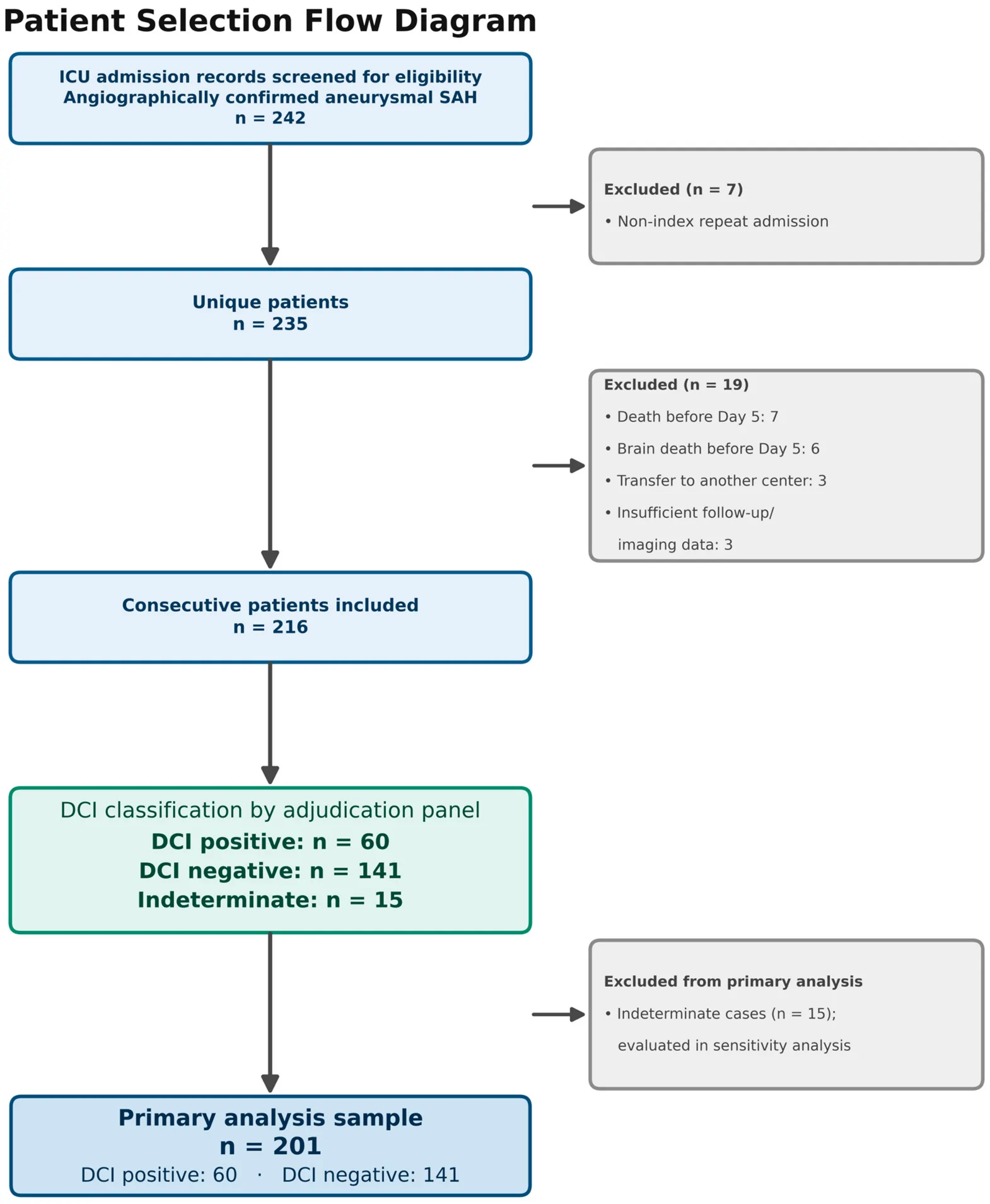
Patient selection flow diagram. A total of 242 ICU admission records with angiographically confirmed aneurysmal subarachnoid hemorrhage (aSAH) were screened; after excluding 7 non-index repeat admissions, 235 unique patients remained, of whom 19 were excluded per the predefined criteria, leaving 216 unique patients included in the analysis. The adjudication panel classified 60 patients as delayed cerebral ischemia (DCI)-positive, 141 as DCI-negative, and 15 as indeterminate; the indeterminate cases were removed from the primary analysis and evaluated in the sensitivity analysis, yielding a primary analysis sample of 201 patients.

During the study period, 242 ICU admission records with angiographically confirmed aSAH were screened for eligibility; 7 records represented non-index repeat admissions and were excluded, leaving 235 unique patients, of whom 19 were further excluded in accordance with the predefined criteria (13 who died or were declared brain-dead before Day 5, 3 transferred to another center before Day 5, and 3 with insufficient follow-up/imaging data), leaving 216 unique patients for analysis (Figure 1). The adjudication panel classified 60 patients (27.8%) as DCI-positive, 141 as DCI-negative, and 15 as indeterminate; after the indeterminate cases were excluded from the primary analysis, the primary sample comprised 201 patients (DCI incidence, 29.9%). The demographic and clinical characteristics of the cohort and their distribution by DCI outcome are presented in Table 1. Apart from admission GCS, which could not be assessed in 23 sedated patients (10.6%), data were complete for all analyzed variables. Among the 60 DCI-positive patients, the adjudicated diagnosis was based on a new cerebral infarction without qualifying clinical deterioration in 33 (55.0%), on clinical deterioration alone (a new focal neurological deficit and/or a ≥2-point GCS decrease, without a corresponding new infarct) in 14 (23.3%), and on a combination of clinical deterioration and new infarction in 13 (21.7%).

Baseline characteristics were available for 16 of the 19 uniquely excluded patients (13 who died or were declared brain-dead before Day 5, and 3 who were transferred to another center before Day 5); data could not be retrieved for the remaining 3 patients excluded for insufficient follow-up/imaging data. Compared with the analyzed cohort (*n* = 216), these 16 patients showed a markedly more severe baseline neurological and radiological profile, including higher WFNS grade (median 4.5 vs. 2.0), modified Fisher scale (median 4.0 vs. 3.0), and SEBES score (median 3.0 vs. 2.0), and lower admission GCS (median 6.5 vs. 15.0), whereas age and sex distributions were similar (Supplementary Table S5).

The WFNS grade, modified Fisher scale, and SEBES score—markers of baseline disease severity—together with the admission GCS indicated a significantly greater disease burden in patients who developed DCI (all *p* < 0.001). Intraventricular hemorrhage (58.3% vs. 34.8%; *p* = 0.003) and hydrocephalus (20.0% vs. 8.5%; *p* = 0.039) were more frequent in the DCI-positive group. No significant between-group differences were observed in demographic variables or comorbidities.

### 3.2. Secondary Clinical Outcomes

Secondary clinical outcomes were evaluated in the primary sample (*n* = 201) according to DCI outcome. ICU mortality was significantly higher in DCI-positive patients (45.0% vs. 14.9%; *p* < 0.001; 23.9% in the primary analysis sample). ICU length of stay was markedly longer in the DCI-positive group (median 13 [IQR 9–25] days vs. 5 [4,5,6,7,8,9] days; *p* < 0.001). The need for decompressive surgery was more frequent in the DCI-positive group (31.7% vs. 8.5%; *p* < 0.001). The presence of vasospasm did not differ significantly between the two groups (15.0% vs. 13.5%; *p* = 0.950); however, because vasospasm assessment relied on clinical and imaging records rather than on protocolized systematic screening, this finding should be interpreted as descriptive.

### 3.3. Primary Outcome: Discrimination and Non-Inferiority

**Figure 2:**
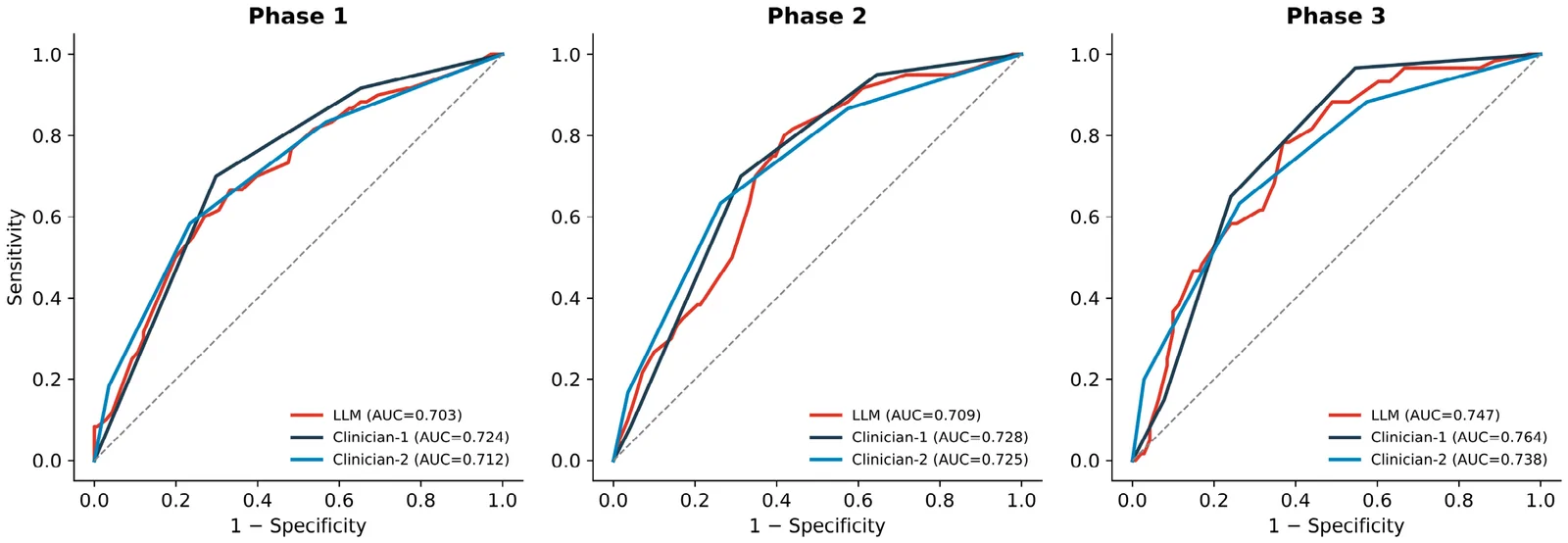
Receiver operating characteristic (ROC) curves for the large language model (LLM) and the two individual clinicians across the three phases; area under the curve (AUC) values are given in the legend. The expert-average curve is omitted for readability; the corresponding AUC values are provided in Table 2A.

DCI discrimination was assessed by AUC across all three evaluation phases (Table 2A; Figure 2). The LLM AUCs were 0.703 in Phase 1, 0.709 in Phase 2, and 0.747 in Phase 3, whereas clinician AUCs ranged from 0.712 to 0.764. The primary comparisons were prespecified as separate comparisons of the LLM with each individual clinician, with the comparison against the expert average prespecified as a supporting analysis.

In the prespecified primary analysis using the native probability outputs, the LLM met the non-inferiority criterion (Δ = 0.10) relative to both clinicians across all three phases: the lower bound of the 95% CI for every AUC difference exceeded −0.10 (Table 2B; Figure S2). This conclusion was subsequently qualified by the equal-granularity sensitivity analysis, in which non-inferiority was not demonstrated in Phase 1 (see below). The differences between the LLM and the individual clinicians were small and remained within the non-inferiority margin. In the supporting comparison against the expert average, non-inferiority was likewise maintained; a small difference was detected only in Phase 1 on the two-sided superiority test (ΔAUC = −0.038; *p* = 0.049), although the non-inferiority criterion was still met. These findings indicate that the LLM’s performance was comparable (non-inferior) to, but not superior to, that of two experienced intensive care specialists.

### 3.4. Dynamic Change Across Phases

**Figure 3:**
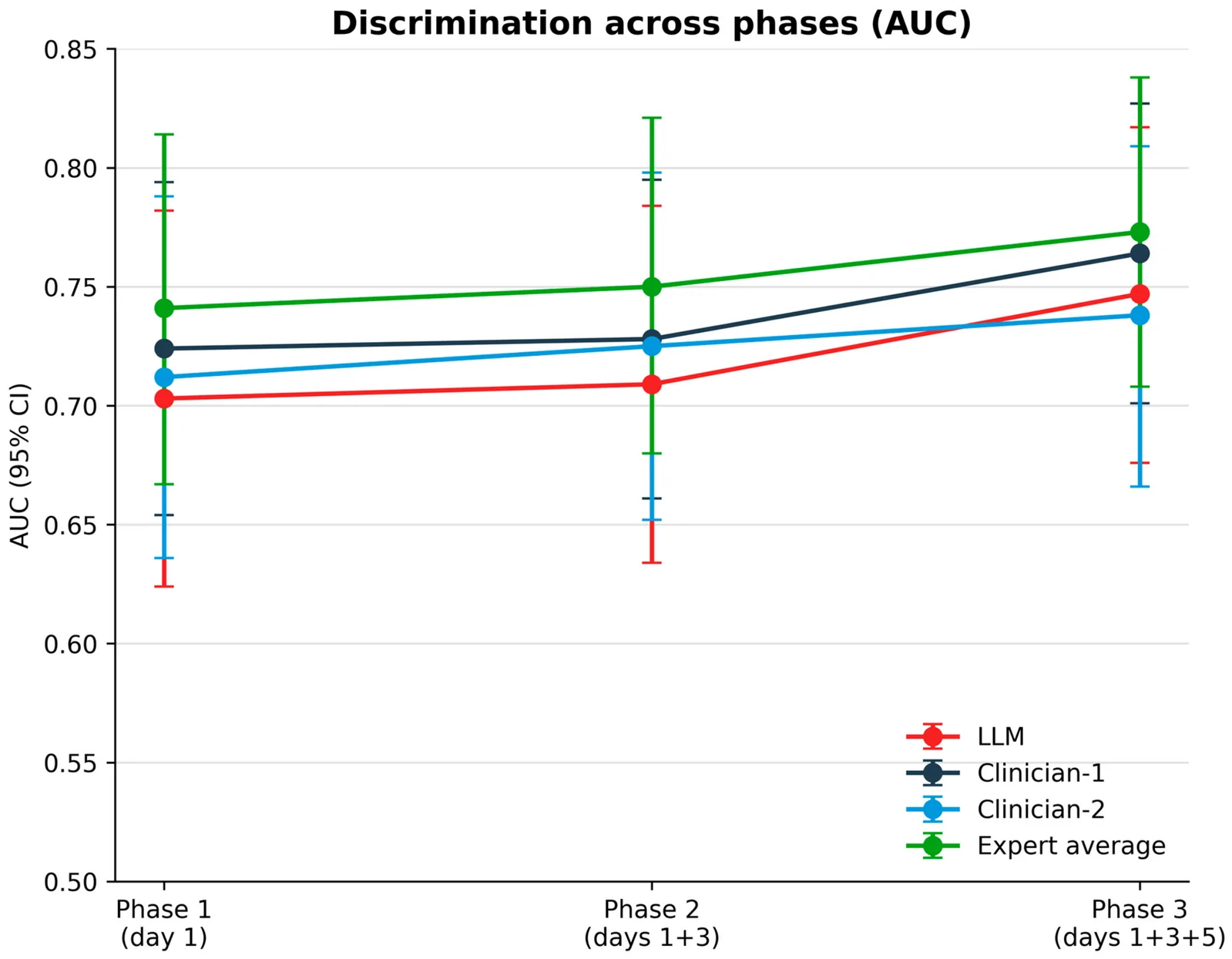
Trajectory of area under the curve (AUC) estimates across phases; error bars denote 95% confidence intervals (CI). The plot is descriptive.

As clinical data accumulated (Day 1 → Days 1 + 3 → Days 1 + 3 + 5), discrimination increased numerically for all assessors (Figure 3): from 0.703 to 0.747 for the LLM, from 0.724 to 0.764 for Clinician 1, and from 0.741 to 0.773 for the expert average. However, in the paired comparison this increase for the LLM did not reach statistical significance (Phase 1 vs. Phase 3 ΔAUC = +0.044; 95% CI −0.004 to +0.091; DeLong *p* = 0.072). This pattern suggests that discrimination rose numerically as clinical data accumulated over time; however, this numerical increase was substantially attenuated in the landmark-restricted sensitivity analysis described below.

### 3.5. Classification, Calibration, and Decision-Curve Analysis

In the secondary, descriptive classification analysis, the LLM’s continuous probability output was dichotomized at the Youden-optimal threshold for each phase (Table 3). Sensitivity ranged from 0.67 to 0.80 and specificity from 0.58 to 0.67. The NPV was relatively high across all phases (0.82–0.87), suggesting that the model’s low-risk classification may have potential value as a supportive low-risk signal rather than a standalone rule-out decision. The PPV was modest (0.45–0.47), reflecting the relatively low DCI prevalence (29.9%) in the primary analysis sample. Calibration was assessed for the LLM using the Brier score (0.183–0.189) and quintile-based calibration curves (Figure S1); visual calibration appeared closest to the ideal line in Phase 1 and showed a slight tendency toward overestimation in the later phases. Formal calibration statistics were consistent with this pattern (Table 4): calibration intercepts became progressively more negative across phases (−0.037, −0.240, and −0.348 for Phases 1–3), with Phase 3 showing modest systematic overprediction (95% CI, −0.677 to −0.019). Calibration slopes did not show a statistically clear deviation from the ideal value of 1 in any phase (0.807, 0.897, and 0.969, respectively). Because the clinician probabilities were derived from the prespecified representative values of the ranges, clinician calibration was reported as exploratory.

Clinical utility was assessed, as an exploratory analysis without external validation, using net-benefit-based DCA (Figure S3). Across all three phases, the LLM provided positive net benefit relative to the “treat-all-as-high-risk” and “treat-none-as-high-risk” strategies, particularly in the low-to-moderate threshold-probability range, and largely overlapped with the clinician curves.

### 3.6. Clinical Safety and Reliability

Only a weak-to-moderate relationship was found between the confidence level reported by the LLM and prediction accuracy. Mean confidence was nearly identical for correct and incorrect predictions (correct, 74.0–74.6; incorrect, 73.0–73.1); by contrast, the error rate was lower among high-confidence predictions (confidence ≥ 75) than among low-confidence ones (Table S2). Nevertheless, approximately one-quarter of the high-confidence predictions were incorrect, so high confidence was not a reliable guarantee of accuracy.

False reassurance—a clinically critical type of error, defined as assigning low risk to a patient who developed DCI—was evaluated by phase (Table 5). The LLM had the lowest false-reassurance rate in all three phases, and this rate declined as the phases progressed (11.7% → 5.0% → 3.3%); the corresponding rates were 11.7–15.0% for Clinician 1 and 16.7–23.3% for Clinician 2. The pattern underlying this difference is that the LLM operated at a more cautious risk threshold: whereas it assigned “low” risk in only 21.8% of all evaluations (vs. ~40% for the clinicians), it also assigned “low” risk less often in patients who did not develop DCI (30.5% vs. ~53%; Table S4). The LLM’s low false-reassurance rate therefore reflected a more cautious—and mildly overestimating—calibration rather than superior discrimination, and it entailed an increased tendency toward over-triage in exchange for a lower risk of missed cases.

The between-run stability of the LLM outputs was assessed across three independent runs in 20 patients (Table 6). Agreement for the risk category was excellent (Fleiss κ = 0.887), and the continuous probability output showed very high reproducibility (ICC = 0.968; mean absolute difference, 3.9 points). The clinical-domain κ values ranged from 0.499 to 0.847, and the reproducibility of the confidence level was moderate (ICC = 0.527). In a phase-specific sensitivity analysis, risk-category Fleiss κ was 0.945, 0.824, and 0.886, and probability ICC was 0.972, 0.964, and 0.968 for Phases 1, 2, and 3, respectively. Phase-specific estimates were consistent with the pooled estimates.

### 3.7. Clinical Reasoning: Domain-Specific Agreement

Although agreement on the overall risk category was moderate-to-substantial (weighted κ 0.628–0.685 between the two specialists and 0.547–0.775 for LLM vs. clinician), domain-specific agreement across the five clinical evaluation domains was markedly lower (LLM–Clinician 1 Cohen’s κ, 0.02–0.36; LLM–Clinician 2, −0.01–0.23), with patient-clustered bootstrap 95% confidence intervals that excluded or approached zero for most domains (Table S1). This exploratory finding indicates that the LLM and the clinicians reached a similar overall risk outcome largely through different domain attributions; however, this pattern should be interpreted in light of the limited measurement reliability of the three-category domain-attribution instrument and the low clinician–clinician domain agreement observed, and it should not be taken as direct evidence that the underlying reasoning structures diverge.

### 3.8. Sensitivity Analysis

The effect of including the 15 cases with an indeterminate adjudication result was evaluated across all three phases (Table S3). These patients represented the most severely affected subgroup of the cohort (median WFNS grade 5.0, median admission GCS 3.0, intraventricular hemorrhage in 93.3%); the documented reason for indeterminate classification was brain death in 7 cases and systemic mortality precluding further neurological assessment in 8 cases (Table S6). When these cases were treated as DCI-positive or DCI-negative in the extreme scenarios, the LLM AUC varied by up to 0.055 from the primary estimate across the extreme indeterminate-handling scenarios (e.g., Phase 3: primary 0.747; indeterminate = positive 0.782; indeterminate = negative 0.692), and the LLM’s discrimination remained above 0.65 in every phase under both extreme scenarios. Clinician AUCs and the corresponding non-inferiority comparisons were not recomputed under these scenarios, so this sensitivity analysis speaks only to the stability of the LLM’s own discrimination and not to the non-inferiority conclusion itself.

### 3.9. Landmark-Restricted Sensitivity Analysis (DCI Onset Timing)

Because the exact calendar day of DCI onset was not captured as a discrete variable in the primary dataset, the adjudication panel subsequently reconstructed this day for all 60 DCI-positive patients from source clinical documentation. Three patients had documented DCI evidence by Day 3 and were excluded from a Phase 2 landmark-restricted cohort (*n* = 198; 57 DCI-positive, 141 DCI-negative); 16 patients had documented DCI evidence by Day 5 and were excluded from a Phase 3 landmark-restricted cohort (*n* = 185; 44 DCI-positive, 141 DCI-negative). The event rate was 28.8% (57/198) in the Phase 2 landmark-restricted cohort and 23.8% (44/185) in the Phase 3 cohort, compared with 29.9% (60/201) in the primary analysis sample. Because all DCI-negative patients were retained while DCI-positive patients with early documented onset were excluded, outcome prevalence decreased as the landmark was extended; differences from the primary analysis therefore reflect both reduced outcome leakage and a change in cohort composition. In these landmark-restricted cohorts, LLM discrimination was materially unchanged relative to the primary estimates (Phase 2: AUC 0.705 vs. 0.709; Phase 3: AUC 0.710 vs. 0.747), and non-inferiority to both clinicians remained supported, with all lower 95% confidence bounds for the paired AUC differences exceeding the prespecified non-inferiority boundary of −0.10 (Phase 2 landmark cohort: LLM–Clinician 1, −0.018 [95% CI, −0.066 to 0.030]; LLM–Clinician 2, −0.011 [95% CI, −0.066 to 0.043]; Phase 3 landmark cohort: LLM–Clinician 1, −0.018 [95% CI, −0.065 to 0.028]; LLM–Clinician 2, +0.006 [95% CI, −0.049 to 0.061]). To distinguish the effect of outcome leakage from that of the accompanying change in case mix, discrimination was further recomputed for all three phases within the single common cohort of 185 patients without documented DCI by Day 5. In this common cohort, LLM AUC was 0.706, 0.700, and 0.710 for Phases 1, 2, and 3, respectively, and the Phase 3 versus Phase 1 paired AUC difference was +0.004 (95% CI, −0.034 to 0.048). Thus, when phase-wise discrimination was compared within the same, landmark-restricted patient population, the phase-related increase observed in the primary analysis was substantially attenuated and no longer distinguishable from no change.

### 3.10. Equal-Granularity Sensitivity Analysis

To address the difference in output granularity between the continuous LLM probabilities and the clinicians’ four probability ranges, an equal-granularity sensitivity analysis was performed by categorizing the LLM probabilities into the same four intervals used by the clinicians. The corresponding LLM AUCs were 0.666 (95% CI, 0.587–0.739), 0.688 (0.618–0.757), and 0.724 (0.655–0.790) for Phases 1–3, respectively, compared with 0.703, 0.709, and 0.747 in the primary continuous-probability analysis. Non-inferiority remained supported in Phase 2 (LLM–Clinician 1, −0.040 [95% CI, −0.098 to 0.013]; LLM–Clinician 2, −0.036 [−0.098 to 0.023]) and Phase 3 (−0.040 [−0.086 to 0.012]; −0.014 [−0.069 to 0.041]). In Phase 1, however, non-inferiority was not demonstrated relative to either Clinician 1 (AUC difference, −0.058; 95% CI, −0.109 to −0.012) or Clinician 2 (−0.046; 95% CI, −0.105 to 0.010), because the lower confidence bounds crossed the prespecified −0.10 margin. These results indicate that the primary findings were partly sensitive to differences in response resolution, particularly in Phase 1.

### 3.11. Exploratory Benchmark Comparison with Established Severity Scores

In an exploratory benchmark analysis, discrimination of the established baseline scores was 0.683 (95% CI, 0.604–0.761) for WFNS grade, 0.671 (0.591–0.751) for the modified Fisher scale, and 0.716 (0.640–0.792) for SEBES. Phase-specific LLM AUCs were 0.703, 0.709, and 0.747 for Phases 1–3, respectively. Across the nine paired comparisons, the LLM did not demonstrate consistently greater discrimination than the established scores (Supplementary Table S8). At Phase 3, the LLM AUC was nominally higher than that of the modified Fisher scale (ΔAUC, +0.076; 95% CI, +0.015 to +0.136; nominal *p* = 0.015), whereas the difference versus WFNS grade did not reach nominal statistical significance (ΔAUC, +0.064; 95% CI, −0.0004 to +0.128; *p* = 0.051). No phase-specific LLM comparison with SEBES reached nominal statistical significance.

## 4. Discussion

In this study, using a phase-based design in 216 consecutive patients with aSAH, the DCI discrimination of a large language model (ChatGPT, GPT-5.5 Thinking) was directly compared with that of two independent, blinded intensive care specialists. The principal findings can be summarized along four lines: (i) the LLM showed discrimination non-inferior to that of two experienced intensive care specialists across all three evaluation phases (Δ = 0.10); (ii) discrimination increased numerically as clinical data accumulated, although this increase did not reach statistical significance; (iii) the LLM exhibited a lower false-reassurance rate, but this stemmed from a more cautious risk classification rather than superior discrimination and was offset by an increased tendency toward over-triage; and (iv) although the model’s confidence level did not reliably reflect accuracy, its probability and risk-category outputs showed high reproducibility. Taken together, these findings indicate that the LLM should be positioned not as an independent decision-maker but as a structured clinical decision-support tool under human supervision.

In the existing literature, the clinical evaluation of language models has largely relied on static, single-time-point, vignette-based designs [12,13]. LLM applications that perform dynamic risk prediction from real clinical records are only beginning to emerge [26,27]. To our knowledge, this is among the first studies to evaluate a phase-based, dynamic LLM–clinician comparison in real, consecutive patients with aSAH, using an adjudicated outcome, blinded expert comparators, and clinical data accumulating over time. Conducting such a comparison—particularly in the intensive care setting and for an outcome as difficult to predict and as clinically critical as DCI—represents the principal novelty of this study and addresses a previously underexplored question: how language-model performance changes over time.

The level of discrimination achieved by the LLM (AUC ~0.70–0.75) is meaningful when one considers that DCI prediction is inherently difficult and that even established clinical scores provide only modest discrimination [7,8]. Although the model’s attainment of performance comparable to experienced specialists is noteworthy, it did not demonstrate superiority. This pattern is consistent with the broader literature showing that LLMs are generally non-inferior to physicians yet may fall short of expert performance [28]. The study’s prespecified non-inferiority framework enabled a cautious and defensible interpretation of the findings while avoiding an inflated superiority claim; even the small difference detected against the expert average in Phase 1 remained within the non-inferiority margin.

Among the three established baseline measures, SEBES showed the highest discrimination. The LLM did not significantly outperform SEBES at any phase, including Phase 3. These exploratory benchmark comparisons suggest that the LLM’s discrimination was broadly within the range of established aSAH severity scores rather than uniformly superior to them. The nominally higher Phase 3 discrimination relative to the modified Fisher scale should be interpreted cautiously because these exploratory comparisons were not adjusted for multiplicity. This comparison should also be interpreted with the structural difference between the predictors in mind: WFNS grade, the modified Fisher scale, and SEBES are static baseline measures obtained at admission, whereas the phase-specific LLM predictions incorporated accumulating longitudinal clinical information. The benchmark therefore provides a contextual comparison rather than a like-for-like comparison of predictors using identical information.

A numerical increase in discrimination as clinical data accumulated (Day 1 → Days 1 + 3 → Days 1 + 3 + 5) was observed in the primary analysis; however, this increase did not reach statistical significance in the paired Phase 1–Phase 3 comparison (*p* = 0.072). A landmark-restricted sensitivity analysis that reconstructed DCI onset timing and held the analyzed patient population constant across phases showed that this apparent phase-related increase was substantially attenuated (Phase 3 vs. Phase 1 AUC difference, +0.004; 95% CI, −0.034 to 0.048). This pattern suggests that the numerical increase observed in the primary analysis may be explained, at least in part, by outcome leakage—i.e., some Phase 2 and Phase 3 predictor variables being recorded after DCI onset—rather than by a genuine improvement in predictive performance as clinical information accumulates. In addition, an equal-granularity sensitivity analysis indicated that the primary non-inferiority findings were partly sensitive to differences in response resolution: although non-inferiority remained supported in Phases 2 and 3, it was not demonstrated in Phase 1 after the LLM probabilities were reduced to the same four-category resolution used by the clinicians. This reinforces the need for caution when comparing continuous model outputs with coarser clinician probability estimates. These interpretations should be confirmed in future prospective studies with daily-resolution outcome ascertainment.

In the calibration analysis, the LLM’s probability estimates showed limited but acceptable agreement; the slight tendency toward overestimation observed in the later phases is consistent with a tendency of the model to estimate risk probabilities upward. The exploratory decision-curve analysis showed that the LLM provided positive net benefit and that its net-benefit curves largely overlapped with those of the clinicians over the low-to-moderate threshold range in this cohort. However, because these findings were internally derived and were not externally validated, they do not establish clinical utility and should be interpreted as hypothesis-generating within an investigational decision-support framework.

One notable observation in this study concerns the relationship between false-reassurance rates and the decision threshold. The LLM showed a lower rate of assigning low risk to patients who developed DCI (false reassurance) than the clinicians, and this rate decreased as the phases progressed. However, this advantage arose not from superior discrimination but from the model operating at a systematically more cautious risk threshold: the LLM assigned “low” risk markedly less often than the clinicians, both overall and in patients who did not develop DCI. The lower rate of low-risk assignment in patients who developed DCI was therefore accompanied by an increased tendency toward over-triage. This trade-off is context-dependent: for a serious and treatable complication such as DCI, a cautious approach may be acceptable, but unnecessary advanced imaging and intensive monitoring may impose a resource and cost burden. Although the relatively high NPV suggests that the model’s low-risk classification may have potential in resource-limited settings—not as a standalone rule-out decision but as a clinician-supervised prioritization tool or low-risk supportive signal—this possible role must be tested prospectively. While the LLM and one clinician showed a progressive decline in false-reassurance rate across the phases, the rate for the other clinician followed a non-monotonic course; however, given the relatively small number of DCI events, these phase-level differences most likely reflect sampling variability.

The fact that the model’s reported confidence level reflected prediction accuracy only weakly is an important caveat for clinical use. Although high-confidence predictions were less often incorrect than low-confidence ones, approximately one-quarter of the high-confidence predictions were nonetheless wrong. The model’s confidence score should therefore not be used as an independent, reliable indicator of the accuracy of a prediction [16,21,22]; this reinforces the conclusion that LLM outputs require human oversight. Model-reported confidence should therefore not be interpreted as a validated reliability signal. Displaying such values to clinicians could potentially promote unwarranted trust if their meaning is not clearly qualified. Whether confidence scores should be displayed, suppressed, or contextualized in future clinical interfaces requires prospective human–AI interaction studies.

The high between-run reproducibility of the LLM outputs (for both risk category and probability) is reassuring, as it indicates that the model can produce consistent and reproducible decisions. By contrast, the low domain-specific agreement across the five clinical evaluation domains suggests that the LLM and the clinicians may arrive at similar overall risk outcomes through different domain attributions. However, this exploratory finding should be interpreted in light of the limited measurement reliability of the three-category domain-attribution instrument and the low clinician–clinician domain agreement observed; it should therefore not be taken as direct evidence of a mechanistic divergence in reasoning [16,29].

Taken together, these findings present a balanced picture of the potential role of language models in intensive care. The LLM exhibited strengths such as discrimination non-inferior to that of the clinicians, high reproducibility, and a low rate of low-risk assignment in patients who developed DCI, while carrying limitations including a modest PPV, a tendency toward over-triage, an unreliable confidence score, and limited agreement in domain attribution. This profile supports positioning the model not as an autonomous diagnostic or decision-making tool but as a structured decision-support layer whose outputs are ultimately verified by a clinician [14].

## 5. Limitations

This study has several limitations. First, it has a single-center, retrospective design; the generalizability of the findings should be confirmed in multicenter, prospective studies [30]. No external validation was performed; therefore, the observed performance estimates remain internally derived and require confirmation in independent cohorts. The high proportion of surgical clipping in the cohort may limit the generalizability of the results to centers where endovascular treatment predominates. Furthermore, the prompts were administered in Turkish, and the model inputs reflected the documentation practices and clinical workflow of a single Turkish tertiary ICU. Performance may differ when prompts are translated or administered in other languages, and the present findings therefore should not be assumed to generalize directly across linguistic settings. Similarly, differences between institutions and healthcare systems in documentation standards, monitoring protocols, imaging availability, and the timing of clinical assessments may alter the information presented to the model and consequently its predictive performance. External validation across languages, institutions, and healthcare systems is therefore required. Second, because the study was based on a phase-based landmark design, the analyzed cohort was conditional on day-5 survival and did not include severe cases lost before day 5 (a competing-risk subgroup for DCI). These patients died, were declared brain-dead, or were transferred before completion of the Day-5 landmark and therefore could not undergo the complete three-phase assessment required by the prespecified study design; their DCI status was not adjudicated within the full study framework. A descriptive comparison of the 16 of 19 uniquely excluded patients with retrievable baseline data confirmed a markedly more severe baseline neurological and radiological profile relative to the analyzed cohort (Supplementary Table S5). Because high WFNS, modified Fisher, and SEBES scores are established DCI risk factors, and because early death represents a competing risk for DCI, this survivor-conditioned sampling may affect the observed DCI incidence (27.8%) and limit generalizability to the full spectrum of aSAH severity; the analyzed cohort underrepresents the most severe early presentations. The findings should therefore be generalized primarily to patients who pass the day-5 landmark and remain under follow-up; accordingly, these findings should not be extrapolated to the sickest aSAH patients with early mortality or early neurological death before the day-5 landmark. Nevertheless, because the LLM and the clinicians were evaluated on exactly the same cohort, this selection limits external validity but does not affect the internal validity of the comparison. Third, no a priori sample-size calculation was performed (see Statistical analysis); the available sample yielded reasonably precise estimates of the primary AUC differences but offered limited power for testing between-phase differences. With 60 DCI events, the confidence intervals around the paired AUC differences remained relatively wide, limiting the precision with which small between-assessor differences could be resolved. The non-inferiority findings should therefore be interpreted in the context of this uncertainty. The limited event count also restricted the precision of secondary and exploratory analyses, which should be considered hypothesis-generating. Fourth, the LLM was used through the web interface for the purpose of ecological validity; this approach introduces limitations such as model-version drift and non-deterministic outputs. Because the LLM was accessed through the commercial web interface, backend generation parameters, including temperature, were not user-configurable. The reproducibility analysis characterized short-term output variability under the study conditions but cannot address future model-version drift. Fifth, because the clinician probability estimates were derived from the prespecified representative values of the structured ranges, clinician calibration can be interpreted only as exploratory; moreover, the LLM probability outputs were recorded as continuous percentages whereas the clinician estimates were recorded as four probability ranges, and the resulting difference in output resolution—which produces substantially more tied values among the clinician estimates—can affect rank-based discrimination measures. An equal-granularity sensitivity analysis (see Results) showed that the primary findings were partly sensitive to this asymmetry: non-inferiority remained supported in Phases 2 and 3 but was not demonstrated in Phase 1 once the LLM probabilities were reduced to the same four-category resolution used by the clinicians. Sixth, the domain-specific agreement analyses pooled the three phases and were therefore interpreted as exploratory; within-patient dependence was addressed using patient-level clustered bootstrap confidence intervals. Seventh, not explicitly disclosing the formal study phase to the assessors strengthened information symmetry between the LLM and the clinicians but partly diverges from the real clinical context. Eighth, the exact calendar day of DCI onset was not captured as a discrete variable in the primary dataset. For the revision, the adjudication panel reconstructed this day from source clinical documentation for all 60 DCI-positive patients, enabling a landmark-restricted sensitivity analysis (see Results). Non-inferiority to both clinicians was preserved after excluding patients with DCI documented by the relevant phase landmark, and, when phase-wise discrimination was compared within a single common cohort of patients without documented DCI by Day 5, the phase-related increase observed in the primary analysis was substantially attenuated. This indicates that part of the numerical increase reported in the primary analysis likely reflected outcome leakage rather than a genuine improvement in predictive performance as clinical information accumulated. Prospective studies should record DCI onset at daily resolution from the outset. In addition, the structured LLM outputs were manually transcribed into the study database by a single investigator without independent second-investigator verification, introducing a potential risk of transcription error despite retention of the original records for auditability. A further consideration is partial circularity between the predictor set and outcome ascertainment, because serial GCS values were provided as phase-specific inputs while a ≥2-point GCS decrease was also one criterion for clinical DCI. Because both assessor groups received the same serial GCS values, this overlap may have inflated absolute discrimination estimates for all assessors by allowing emerging outcome information to contribute to prediction, rather than selectively favoring the LLM. Accordingly, true prospective predictive performance may be somewhat lower when all predictor information is fixed strictly before the earliest qualifying DCI evidence and outcome-defining information is prevented from entering the prediction window. Finally, the results pertain to the specific ChatGPT interface-displayed model evaluated under the study conditions and during a specific time period. They require external validation and replication with other models and should not be generalized to large language models as a class.

## 6. Conclusions

In this phase-based comparative study of patients with aSAH, the prespecified primary analysis supported non-inferiority of the large language model relative to two experienced intensive care specialists in DCI discrimination across all three phases. This finding was, however, partly sensitive to output granularity: non-inferiority was not demonstrated in Phase 1 when the LLM probabilities were reduced to the same four-category resolution used by the clinicians. A numerical increase in discrimination with accumulating clinical data was observed in the primary analysis but was substantially attenuated in a landmark-restricted sensitivity analysis that reconstructed DCI onset timing. Although the model showed high output reproducibility, its lower false-reassurance rate reflected a more cautious, over-triage-prone risk classification rather than a demonstrated safety benefit, and the model’s confidence score was not a reliable indicator of accuracy. The language model should therefore be regarded not as an independent decision-maker but as a structured clinical decision-support tool whose outputs are verified under clinician supervision. These findings apply to the specific ChatGPT model evaluated under the study conditions described and should not be generalized to large language models as a class. Prospective, multicenter studies with external validation are needed before these findings can be translated into clinical practice.

## Figures and Tables

**Table 1:** Patient characteristics and distribution by DCI outcome. Continuous variables are presented as mean ± SD or median (IQR) according to distribution; categorical variables as n (%). *p* values are for the comparison between the DCI-positive and DCI-negative groups; indeterminate cases (*n* = 15) are included only in the Overall column. DCI, delayed cerebral ischemia; GCS, Glasgow Coma Scale; EVD, external ventricular drainage; IQR, interquartile range; SD, standard deviation; SEBES, Subarachnoid Hemorrhage Early Brain Edema Score; WFNS, World Federation of Neurosurgical Societies. Admission-to-procedure time was calculated only among patients who underwent aneurysm-securing treatment (coiling or clipping; *n* = 204); it does not apply to the 12 conservatively managed patients, for whom no aneurysm-securing procedure was performed and the reason was not systematically recorded.

Variable	Overall (*n* = 216)	DCI-Positive (*n* = 60)	DCI-Negative (*n* = 141)	*p*
Age, years	55.8 ± 13.4	58.8 ± 12.2	55.0 ± 13.7	0.053
Sex, female	109 (50.5)	31 (51.7)	73 (51.8)	1.000
Admission-to-procedure time, days	1.0 (0.0–1.0)	1.0 (0.0–1.0)	1.0 (0.0–1.0)	0.469
Hypertension	88 (40.7)	24 (40.0)	59 (41.8)	0.931
Diabetes mellitus	25 (11.6)	10 (16.7)	13 (9.2)	0.202
Coronary artery disease	14 (6.5)	4 (6.7)	8 (5.7)	0.754
Other comorbidity	29 (13.4)	6 (10.0)	20 (14.2)	0.562
WFNS grade (median)	2.0 (1.0–4.0)	3.5 (1.0–4.0)	1.0 (1.0–3.0)	<0.001
Modified Fisher scale (median)	3.0 (1.0–4.0)	3.5 (2.0–4.0)	2.0 (1.0–3.0)	<0.001
SEBES score (median)	2.0 (1.0–3.0)	3.0 (2.0–3.0)	2.0 (1.0–2.0)	<0.001
Admission GCS (evaluable)	15.0 (12.0–15.0)	14.0 (9.0–15.0)	15.0 (14.0–15.0)	<0.001
Sedated/not evaluable at admission	23 (10.6)	9 (15.0)	8 (5.7)	0.058
Intraparenchymal hematoma	62 (28.7)	21 (35.0)	33 (23.4)	0.128
Intraventricular hemorrhage	98 (45.4)	35 (58.3)	49 (34.8)	0.003
Hydrocephalus	27 (12.5)	12 (20.0)	12 (8.5)	0.039
Midline shift	28 (13.0)	8 (13.3)	13 (9.2)	0.535
Multiple aneurysms	43 (19.9)	17 (28.3)	25 (17.7)	0.133
Treatment modality				0.195
Coiling	37 (17.1)	6 (10.0)	29 (20.6)	
Clipping	167 (77.3)	51 (85.0)	106 (75.2)	
None/conservative	12 (5.6)	3 (5.0)	6 (4.3)	
EVD	26 (12.0)	11 (18.3)	11 (7.8)	0.052
Nimodipine	215 (99.5)	59 (98.3)	141 (100.0)	0.299
Antithrombotic use	27 (12.5)	7 (11.7)	17 (12.1)	1.000

**Table 2:** (A) Phase-wise discrimination (AUC, 95% CI; DeLong). AUC, area under the receiver operating characteristic curve; CI, confidence interval; LLM, large language model. (B) AUC differences between the LLM and the comparators, and non-inferiority assessment (Δ = 0.10). *p* values were calculated with the DeLong test for the two-sided AUC difference (superiority); the non-inferiority decision was based on whether the lower bound of the 95% CI remained above −0.10. AUC, area under the receiver operating characteristic curve; CI, confidence interval; LLM, large language model; NI, non-inferiority met (Δ = 0.10).

**(A)**					
**Assessor**	**Phase 1**	**Phase 2**	**Phase 3**		
LLM	0.703 (0.624–0.782)	0.709 (0.634–0.784)	0.747 (0.676–0.817)		
Clinician 1	0.724 (0.654–0.794)	0.728 (0.661–0.795)	0.764 (0.701–0.827)		
Clinician 2	0.712 (0.636–0.788)	0.725 (0.652–0.798)	0.738 (0.666–0.809)		
Expert average	0.741 (0.667–0.814)	0.750 (0.680–0.821)	0.773 (0.708–0.838)		
**(B)**					
**Phase**	**Comparison**	**ΔAUC**	**95% CI**	***p***	**NI**
Phase 1	LLM–Clinician 1	−0.021	(−0.064 to +0.022)	0.343	Yes
Phase 1	LLM–Clinician 2	−0.009	(−0.057 to +0.038)	0.707	Yes
Phase 1	LLM–Expert average	−0.038	(−0.075 to −0.0002)	0.049	Yes
Phase 2	LLM–Clinician 1	−0.019	(−0.067 to +0.029)	0.434	Yes
Phase 2	LLM–Clinician 2	−0.016	(−0.069 to +0.037)	0.557	Yes
Phase 2	LLM–Expert average	−0.042	(−0.085 to +0.002)	0.062	Yes
Phase 3	LLM–Clinician 1	−0.017	(−0.059 to +0.025)	0.420	Yes
Phase 3	LLM–Clinician 2	+0.009	(−0.039 to +0.057)	0.713	Yes
Phase 3	LLM–Expert average	−0.026	(−0.061 to +0.009)	0.139	Yes

**Table 3:** LLM classification metrics at the Youden-optimal threshold, with 95% confidence intervals. Confidence intervals for sensitivity, specificity, PPV, and NPV were calculated using the Wilson score method. Thresholds were derived from the same dataset in which performance was evaluated; these estimates are therefore apparent (internally derived) values. CI, confidence interval; LLM, large language model; NPV, negative predictive value; PPV, positive predictive value.

Phase	Youden Threshold	Sensitivity (95% CI)	Specificity (95% CI)	PPV (95% CI)	NPV (95% CI)
Phase 1	35%	0.67 (0.54–0.77)	0.67 (0.59–0.74)	0.46 (0.36–0.56)	0.82 (0.74–0.88)
Phase 2	32%	0.80 (0.68–0.88)	0.58 (0.50–0.66)	0.45 (0.36–0.54)	0.87 (0.79–0.93)
Phase 3	35%	0.78 (0.66–0.87)	0.63 (0.55–0.71)	0.47 (0.38–0.57)	0.87 (0.79–0.92)

**Table 4:** Formal calibration statistics for the LLM probability outputs, by phase. Calibration intercept and slope were estimated using logistic calibration models relating the observed DCI outcome to the logit of the LLM-predicted probability; ideal calibration corresponds to an intercept of 0 and a slope of 1. A negative intercept indicates systematic overprediction. CI, confidence interval; DCI, delayed cerebral ischemia; LLM, large language model.

Phase	Calibration Intercept (95% CI)	Calibration Slope (95% CI)	Brier Score
Phase 1	−0.037 (−0.363 to 0.290)	0.807 (0.436 to 1.178)	0.187
Phase 2	−0.240 (−0.565 to 0.085)	0.897 (0.505 to 1.289)	0.189
Phase 3	−0.348 (−0.677 to −0.019)	0.969 (0.590 to 1.348)	0.183

**Table 5:** Assignment of “low” risk in DCI-positive patients (false reassurance), by phase. Because the LLM and the clinicians used different probability-output formats, this descriptive comparison was based on each assessor’s native low-risk category; probability ranges were not used to define low risk for this analysis. DCI, delayed cerebral ischemia; LLM, large language model.

Phase	LLM	Clinician 1	Clinician 2
Phase 1	7/60 (11.7%)	9/60 (15.0%)	14/60 (23.3%)
Phase 2	3/60 (5.0%)	7/60 (11.7%)	12/60 (20.0%)
Phase 3	2/60 (3.3%)	9/60 (15.0%)	10/60 (16.7%)

**Table 6:** Reproducibility substudy (20 patients × 3 phases × 3 runs). Confidence intervals were obtained by patient-level clustered bootstrap resampling (2000 resamples; percentile method). The wide interval for the radiological-burden domain reflects the limited variability of this domain within the 20-patient reproducibility substudy. CI, confidence interval; ICC, intraclass correlation coefficient.

Output	Metric	Value (95% CI)
Risk category	Fleiss κ	0.887 (0.751–0.981)
Neurological severity	Fleiss κ	0.750 (0.349–0.958)
Radiological burden	Fleiss κ	0.579 (−0.041–1.000)
Inflammatory response	Fleiss κ	0.847 (0.714–0.960)
Hemodynamic instability	Fleiss κ	0.499 (0.322–0.627)
Metabolic derangement	Fleiss κ	0.638 (0.479–0.763)
Probability	ICC(2,1)	0.968 (0.938–0.978)
Confidence	ICC(2,1)	0.527 (0.290–0.657)

## Data Availability

The data presented in this study are available on request from the corresponding author due to patient privacy and ethical restrictions. A complete data dictionary describing all study variables, their definitions, coding, phase availability, and missing-data handling is provided as Supplementary Table S7. Code reproducing the statistical analyses reported in this study is publicly available at https://doi.org/10.5281/zenodo.22073048 (MIT Licence); patient-level data are not included in that repository. The standardized prompt and the structured adjudication checklist are provided as Supplementary Files S2 and S3. The full audit-trail materials underlying the LLM evaluations (time-stamped PDF records, ChatGPT chat URLs, screenshots, and screen recordings) contain patient-level data and are therefore not publicly deposited; they are retained and available to the editors and reviewers upon reasonable request. The study was not prospectively registered; the analysis framework, phase structure, prompt format, output fields, adjudication process, and non-inferiority margin (Δ = 0.10 AUC) were prespecified before the main model and clinician evaluations.

## Supplementary Materials

The following supporting information can be downloaded at: https://www.mdpi.com/article/10.3390/medicina62091680/s1, Supplementary File S1: Supplementary Tables S1–S8 and Supplementary Figures S1–S3. Supplementary File S2: Standardized prompt used for the LLM evaluation (Turkish original and English translation). Supplementary File S3: Structured adjudication checklist for delayed cerebral ischemia (Turkish original and English translation). Supplementary File S4: TRIPOD-LLM reporting checklist. Supplementary File S5: STROBE reporting checklist.

## Abbreviations

AI, artificial intelligence; aSAH, aneurysmal subarachnoid hemorrhage; AUC, area under the receiver operating characteristic curve; CI, confidence interval; CRP, C-reactive protein; CTA, CT angiography; DCA, decision curve analysis; DCI, delayed cerebral ischemia; DSA, digital subtraction angiography; EVD, external ventricular drainage; GCS, Glasgow Coma Scale; ICC, intraclass correlation coefficient; ICU, intensive care unit; IQR, interquartile range; LLM, large language model; NLR, neutrophil-to-lymphocyte ratio; NPV, negative predictive value; PPV, positive predictive value; ROC, receiver operating characteristic; SD, standard deviation; SEBES, Subarachnoid Hemorrhage Early Brain Edema Score; WFNS, World Federation of Neurosurgical Societies.
