# Supplementary material for: Phase-Based Dynamic Prediction of Delayed Cerebral Ischemia After Aneurysmal Subarachnoid Hemorrhage: Comparison of a Large Language Model with Intensive Care Specialists
Source: Medicina (Kaunas). 2026 Sep 2;62(9):1680. doi: 10.3390/medicina62091680 (PMC13609773; doi:10.3390/medicina62091680)
Supplement: Supplementary file 1 [file medicina-62-01680-s001.zip › Supplementary_File_S1_Tables_Figures.pdf]

Supplementary File S1

Supplementary tables and figures

Phase-Based Dynamic Prediction of Delayed Cerebral Ischemia After Aneurysmal Subarachnoid Hemorrhage: Comparison of a Large Language Model With Intensive Care Specialists

Supplementary Table S1. Domain-specific agreement (Cohen κ with patient-clustered bootstrap 95% CI; three phases pooled, exploratory)

Values were computed by pooling the three phases per patient; because a given patient could contribute up to three correlated observations, 95% confidence intervals were obtained by patient-level clustered bootstrap resampling (2000 resamples), in which all phase-level observations belonging to a resampled patient were kept together. Point estimates should nonetheless be interpreted as exploratory. Because Clinician 2 selected the same category ("supports") for the radiological-burden domain in all 648 evaluations, this domain had zero variance for Clinician 2; κ is therefore mathematically undefined for any comparison involving Clinician 2 in this domain and is shown as "—" rather than as a numerical value.

Clinical domain	LLM–Clinician 1 κ (95% CI)	LLM–Clinician 2 κ (95% CI)	Clinician 1–Clinician 2 κ (95% CI)
Neurological severity	0.075 (0.026–0.128)	−0.009 (−0.024–0.000)	−0.007 (−0.014–0.000)
Radiological burden	0.355 (0.163–0.528)	—	—
Inflammatory response	0.254 (0.202–0.303)	0.054 (0.037–0.075)	0.247 (0.190–0.310)
Hemodynamic instability	0.019 (−0.046–0.088)	0.019 (0.000–0.042)	0.016 (0.004–0.029)
Metabolic derangement	0.316 (0.249–0.388)	0.234 (0.178–0.294)	0.565 (0.469–0.653)

Supplementary Table S2. Error rate by LLM confidence level (Youden threshold)

Phase	High-confidence error rate (confidence ≥ 75)	Low-confidence error rate (confidence < 75)
Phase 1	22/85 (25.9%)	45/116 (38.8%)
Phase 2	17/75 (22.7%)	54/126 (42.9%)
Phase 3	20/72 (27.8%)	45/129 (34.9%)

Supplementary Table S3. Sensitivity analysis for indeterminate cases (LLM AUC)

Phase	Primary (indeterminate excluded)	Indeterminate = DCI-positive	Indeterminate = DCI-negative
Phase 1	0.703	0.749	0.654
Phase 2	0.709	0.753	0.657
Phase 3	0.747	0.782	0.692

Supplementary Table S4. Risk-category usage pattern (cautious threshold / over-triage)

Values are pooled (three-phase) percentages. The LLM used the "low" risk category markedly less often than the clinicians and the "high" risk category more often, supporting the interpretation that its low false-reassurance rate stems from a more cautious operating threshold and an increased tendency toward over-triage.

Assessor	"Low," all (%)	"High," all (%)	"Low" in DCI− (%)	"High" in DCI+ (%)
LLM	21.8	43.7	30.5	62.2
Clinician 1	38.9	29.3	53.7	42.2
Clinician 2	40.9	14.2	53.0	23.9

Supplementary Table S5. Baseline characteristics of the analyzed cohort and evaluable excluded patients

Variable	Analyzed cohort (n = 216)	Evaluable excluded patients (n = 16)
Age, years	55.8 ± 13.4 (mean ± SD)	58.0 (42.8–70.5)
Sex, female	109 (50.5)	8 (50.0)
WFNS grade	2.0 (1.0–4.0)	4.5 (2.5–5.0)
Modified Fisher scale	3.0 (1.0–4.0)	4.0 (2.8–4.0)
SEBES score	2.0 (1.0–3.0)	3.0 (2.0–4.0)
Admission GCS	15.0 (12.0–15.0)	6.5 (3.0–14.0)
Intraventricular hemorrhage	98 (45.4)	12 (75.0)
Hydrocephalus	27 (12.5)	4 (25.0)

Continuous variables are presented as mean ± SD or median (IQR), as indicated; categorical variables are n (%). Baseline data were available for 16 of the 19 uniquely excluded patients (13 who died or were declared brain-dead before Day 5, and 3 transferred to another center before Day 5); data could not be retrieved for the remaining 3 patients excluded for insufficient follow-up/imaging data. Patients excluded for non-index repeat admission (n = 7) are not shown, as their index admission is already represented in the analyzed cohort. Given the small size of the excluded group, no formal statistical comparison was performed; values are presented descriptively. DCI, delayed cerebral ischemia; GCS, Glasgow Coma Scale; IQR, interquartile range; SEBES, Subarachnoid Hemorrhage Early Brain Edema Score; WFNS, World Federation of Neurosurgical Societies.

Supplementary Table S6. Characteristics of patients with an indeterminate DCI adjudication result (n = 15)

Variable	Indeterminate cases (n = 15)
Age, years	48.0 (46.0–58.0)
Sex, female	5 (33.3)
WFNS grade	5.0 (5.0–5.0)
Modified Fisher scale	4.0 (3.5–4.0)
SEBES score	3.0 (3.0–4.0)
Admission GCS	3.0 (1.0–4.0)
Intraventricular hemorrhage	14 (93.3)
Hydrocephalus	3 (20.0)
Reason for indeterminate classification	Brain death, 7 (46.7); systemic mortality, 8 (53.3)

Continuous variables are median (IQR); categorical variables are n (%). Indeterminate classification reflects cases in which the adjudication panel could not reach a definitive DCI determination; the documented reason was brain death in 7 cases and systemic (non-neurological) mortality precluding further neurological assessment in 8 cases. These 15 patients were excluded from the primary analysis and evaluated in the sensitivity analysis (Table S3). No formal statistical comparison was performed; values are presented descriptively. DCI, delayed cerebral ischemia; GCS, Glasgow Coma Scale; IQR, interquartile range; IVH, intraventricular hemorrhage; SEBES, Subarachnoid Hemorrhage Early Brain Edema Score; WFNS, World Federation of Neurosurgical Societies.

Supplementary Table S7. Data dictionary: study variables, definitions, coding, phase availability, and missing-data handling

Variable	Definition	Coding / unit	Phase availability	Missing-data handling
Age	Age at ICU admission	Years	Baseline (all phases)	Complete (0%)

Sex	Biological sex	Female / Male	Baseline (all phases)	Complete (0%)
Hypertension	Documented pre-admission diagnosis	Yes / No	Baseline (all phases)	Complete (0%)
Diabetes mellitus	Documented pre-admission diagnosis	Yes / No	Baseline (all phases)	Complete (0%)
Coronary artery disease	Documented pre-admission diagnosis	Yes / No	Baseline (all phases)	Complete (0%)
Other comorbidity	Any other documented chronic comorbidity	Yes / No	Baseline (all phases)	Complete (0%)
Admission-to-procedure time	Days from emergency admission to aneurysm-securing procedure (derived)	Days	Baseline (all phases)	Not applicable for conservatively managed patients (n = 12)
Aneurysm location	Anatomical location of the ruptured aneurysm	Free text; auto-classified as anterior / posterior circulation	Baseline (all phases)	Complete (0%)
Number of aneurysms	Single or multiple aneurysms	Single / Multiple	Baseline (all phases)	Complete (0%)
Treatment modality	Aneurysm-securing treatment	Coiling / Clipping / None (conservative)	Baseline (all phases)	Complete (0%)
Antithrombotic use	Pre-admission antithrombotic medication	Yes / No (agent recorded separately)	Baseline (all phases)	Complete (0%)
Nimodipine	Nimodipine administration	Yes / No	Baseline (all phases)	Complete (0%)
EVD	External ventricular drainage placement	Yes / No	Baseline (all phases)	Complete (0%)
WFNS grade	WFNS grade at admission	1–5	Baseline (all phases)	Complete (0%)
Modified Fisher scale	Modified Fisher grade on admission CT	1–4	Baseline (all phases)	Complete (0%)
SEBES score	Subarachnoid Hemorrhage Early Brain Edema Score on admission CT	0–4	Baseline (all phases)	Complete (0%)
IVH	Intraventricular hemorrhage on admission CT	Yes / No	Baseline (all phases)	Complete (0%)
Hydrocephalus	Hydrocephalus on admission CT	Yes / No	Baseline (all phases)	Complete (0%)
IPH	Intraparenchymal hematoma on admission CT	Yes / No	Baseline (all phases)	Complete (0%)
Midline shift	Midline shift on admission CT	Yes / No	Baseline (all phases)	Complete (0%)
Admission GCS	GCS at emergency admission	3–15; coded 1 if sedated/not evaluable	Baseline (all phases)	Not evaluable due to sedation in 23 (10.6%); treated as missing in analyses
Pupillary status	Pupillary examination at admission	Isocoric / Anisocoric / Fixed-dilated	Baseline (all phases)	Complete (0%)
GCS (phase)	GCS on the corresponding study day	3–15; coded 1 if sedated/not evaluable	Day 1 / Day 3 / Day 5	Complete (0%)

Sedation	Sedative infusion on the corresponding study day	Yes / No	Day 1 / Day 3 / Day 5	Complete (0%)
Mechanical ventilation	Invasive mechanical ventilation on the corresponding study day	Yes / No	Day 1 / Day 3 / Day 5	Complete (0%)
Vasopressor use	Vasopressor infusion on the corresponding study day	Yes / No	Day 1 / Day 3 / Day 5	Complete (0%)
Infection	Clinically documented infection on the corresponding study day	Yes / No	Day 1 / Day 3 / Day 5	Complete (0%)
Leukocytes (WBC)	White blood cell count	$\times 10^3/mm^3$	Day 1 / Day 3 / Day 5	Complete (0%)
Neutrophils	Absolute neutrophil count	$\times 10^3/mm^3$	Day 1 / Day 3 / Day 5	Complete (0%)
Lymphocytes	Absolute lymphocyte count	$\times 10^3/mm^3$	Day 1 / Day 3 / Day 5	Complete (0%)
NLR	Neutrophil-to-lymphocyte ratio (derived: neutrophils / lymphocytes)	Ratio	Day 1 / Day 3 / Day 5	Complete (0%)
CRP	C-reactive protein	mg/L	Day 1 / Day 3 / Day 5	Complete (0%)
Creatinine	Serum creatinine	mg/dL	Day 1 / Day 3 / Day 5	Complete (0%)
Sodium	Serum sodium	mmol/L	Day 1 / Day 3 / Day 5	Complete (0%)
Glucose	Blood glucose	mg/dL	Day 1 / Day 3 / Day 5	Complete (0%)
Lactate	Arterial lactate	mmol/L	Day 1 / Day 3 / Day 5	Complete (0%)
LLM risk category	LLM-assigned DCI risk category	Low / Moderate / High	Each phase	Complete (0%)
LLM probability	LLM-estimated DCI probability	0–100 (integer %)	Each phase	Complete (0%)
LLM confidence	LLM-reported confidence in its own prediction	0–100 (integer %)	Each phase	Complete (0%)
LLM domain attribution	Contribution of each of five clinical domains to the prediction	Supports / No notable contribution / Inconsistent	Each phase	Complete (0%)
Clinician probability range	Clinician-selected DCI probability range	0–25% / 26–50% / 51–75% / 76–100%; prespecified representative values 12.5 / 37.5 / 62.5 / 87.5 used in continuous analyses	Each phase, each clinician	Complete (0%)
Clinician risk category	Clinician-assigned overall DCI risk category	Low / Moderate / High	Each phase, each clinician	Complete (0%)
Clinician confidence	Clinician-reported confidence	Low (0–33%) / Moderate (34–66%) / High (67–100%)	Each phase, each clinician	Complete (0%)
Clinician domain attribution	Contribution of each of five clinical	Supports / No notable contribution / Inconsistent	Each phase, each clinician	Complete (0%)

	domains to the prediction			
DCI adjudication result	Primary outcome, adjudicated by blinded three-member panel	DCI-positive / DCI-negative / Indeterminate	Outcome	Complete (0%); indeterminate cases (n = 15) excluded from primary analysis
DCI criteria components	New focal deficit; ≥2-point GCS decrease; new attributable infarction; alternative causes excluded	Yes / No for each	Outcome	Complete (0%)
DCI onset day	Study day on which qualifying DCI evidence was first documented (reconstructed for the revision)	Days from hemorrhage	Outcome (DCI-positive only)	Determined for all 60 DCI-positive patients
Indeterminate type	Documented reason for indeterminate adjudication	1 = alive but not neurologically evaluable; 2 = brain death; 3 = systemic mortality	Outcome (indeterminate only)	Complete for all 15 indeterminate cases
ICU length of stay	Duration of ICU stay	Days	Outcome	Complete (0%)
ICU mortality	Death during ICU stay	Yes / No	Outcome	Complete (0%)

Predictor variables designated as baseline or phase-specific were available to both assessors at the indicated phase. Outcome variables were not provided to either assessor. Derived variables were computed automatically within the study database. Laboratory values were the first available result on the corresponding study day. aSAH, aneurysmal subarachnoid hemorrhage; CRP, C-reactive protein; DCI, delayed cerebral ischemia; EVD, external ventricular drainage; GCS, Glasgow Coma Scale; ICU, intensive care unit; IPH, intraparenchymal hematoma; IVH, intraventricular hemorrhage; LLM, large language model; NLR, neutrophil-to-lymphocyte ratio; SEBES, Subarachnoid Hemorrhage Early Brain Edema Score; WBC, white blood cell count; WFNS, World Federation of Neurosurgical Societies.

Supplementary Table S8. Exploratory benchmark comparison of the LLM with established baseline aSAH severity scores

Panel A. Discrimination of each predictor (AUC with 95% CI)

**Predictor**	**AUC (95% CI)**
WFNS grade	0.683 (0.604–0.761)
Modified Fisher scale	0.671 (0.591–0.751)
SEBES score	0.716 (0.640–0.792)
LLM, Phase 1	0.703 (0.624–0.782)
LLM, Phase 2	0.709 (0.634–0.784)
LLM, Phase 3	0.747 (0.676–0.817)

Panel B. Paired comparisons (LLM − baseline score)

**Comparison**	**ΔAUC (95% CI)**	**Nominal p**
LLM Phase 1 − WFNS grade	+0.020 (−0.035 to +0.076)	0.476
LLM Phase 1 − Modified Fisher scale	+0.032 (−0.012 to +0.076)	0.157
LLM Phase 1 − SEBES score	−0.013 (−0.063 to +0.037)	0.609
LLM Phase 2 − WFNS grade	+0.026 (−0.034 to +0.086)	0.393
LLM Phase 2 − Modified Fisher scale	+0.038 (−0.017 to +0.093)	0.180
LLM Phase 2 − SEBES score	−0.007 (−0.067 to +0.053)	0.817
LLM Phase 3 − WFNS grade	+0.064 (−0.0004 to +0.128)	0.051
LLM Phase 3 − Modified Fisher scale	+0.076 (+0.015 to +0.136)	0.015
LLM Phase 3 − SEBES score	+0.031 (−0.038 to +0.099)	0.379

Panel A shows the discrimination of each predictor analysed alone; Panel B shows paired comparisons between each phase-specific LLM output and each baseline score in the same patients. All analyses were performed in the

primary analysis sample (n = 201; 60 DCI events). AUCs and paired differences were computed with the DeLong method. These comparisons were exploratory and nominal two-sided p values are reported without adjustment for multiplicity. AUC, area under the receiver operating characteristic curve; CI, confidence interval; DCI, delayed cerebral ischemia; LLM, large language model; SEBES, Subarachnoid Hemorrhage Early Brain Edema Score; WFNS, World Federation of Neurosurgical Societies.

Supplementary Figures

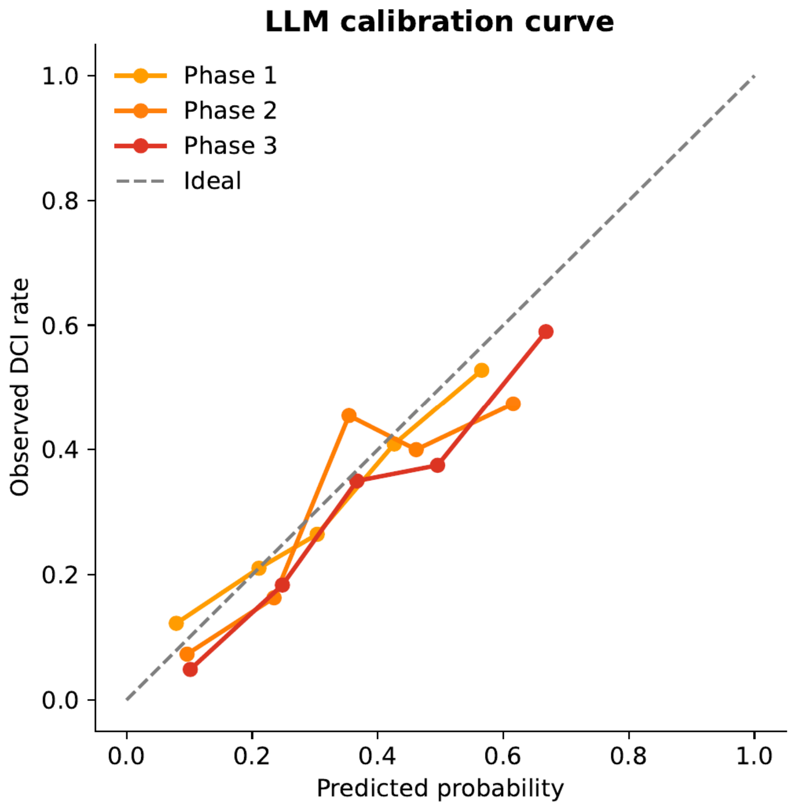

Supplementary Figure S1. LLM calibration curves; the points were formed from equal-frequency (quintile) probability bins, and the dashed line represents ideal calibration.

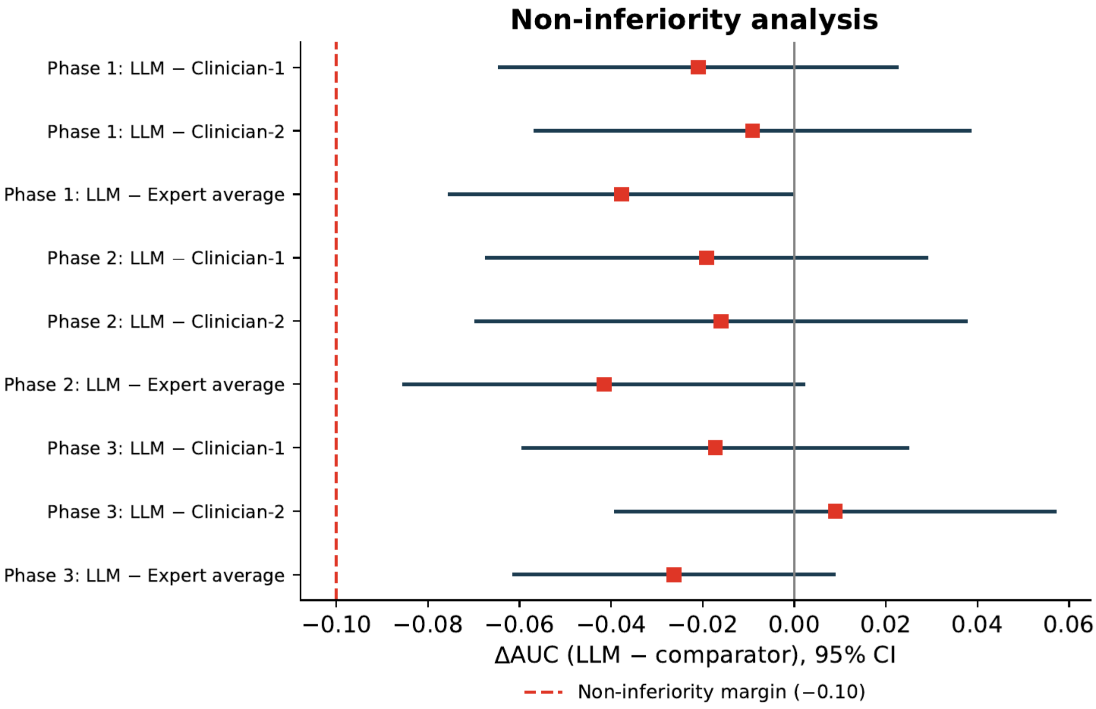

Supplementary Figure S2. Non-inferiority forest plot; the AUC difference between the LLM and the comparators (95% CI) and the prespecified −0.10 margin are shown.

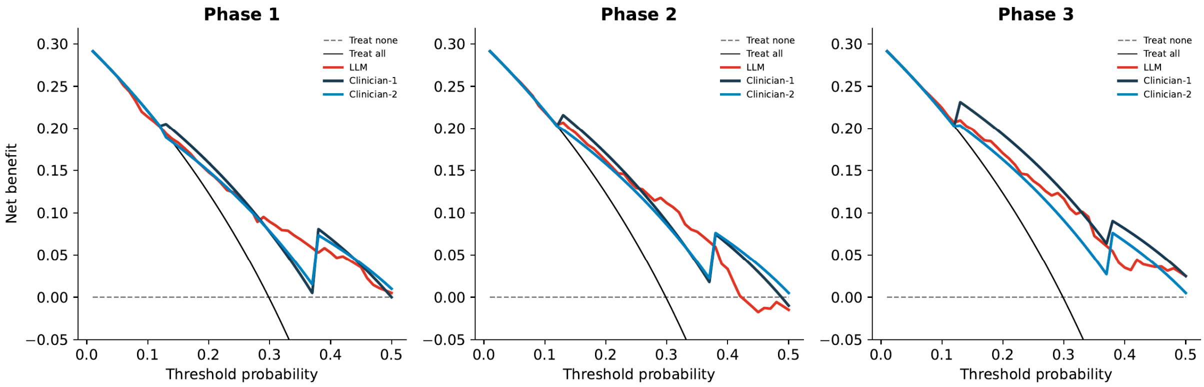

Supplementary Figure S3. Decision curve analysis; net benefit across the three phases is plotted against threshold probability for the LLM, the clinicians, and the "treat-all-as-high-risk" and "treat-none-as-high-risk" strategies.
