# Supplementary material for: Phase-Based Dynamic Prediction of Delayed Cerebral Ischemia After Aneurysmal Subarachnoid Hemorrhage: Comparison of a Large Language Model with Intensive Care Specialists
Source: Medicina (Kaunas). 2026 Sep 2;62(9):1680. doi: 10.3390/medicina62091680 (PMC13609773; doi:10.3390/medicina62091680)
Supplement: Supplementary file 1 [file medicina-62-01680-s001.zip › Supplementary_File_S2_Prompt.pdf]

Supplementary File S2

Standardized prompt used for the LLM evaluation (Turkish original and English translation)

Phase-Based Dynamic Prediction of Delayed Cerebral Ischemia After Aneurysmal Subarachnoid Hemorrhage: Comparison of a Large Language Model With Intensive Care Specialists

The prompt below was applied unchanged to every patient–phase evaluation; only the [HASTA VERİLERİ] / [PATIENT DATA] field was replaced with the data available up to the relevant phase. The Turkish prompt is the version that was actually submitted to the model; the English text is a translation provided for readers and uses the same terminology as the main manuscript. To document the input structure without disclosing any patient data, the fields placed in the [HASTA VERİLERİ] / [PATIENT DATA] block are shown as a schema (field names and units; values left blank). For Phases 2 and 3, the dynamic values of the additional available days (day 3 and day 5) were appended in the same format.

A.1 Turkish prompt — standardized template (as submitted to the model)

```
Anevrizmal subaraknoid kanama (aSAK) konusunda deneyimli bir yoğun bakım uzmanı
olarak değerlendir.

Bu hasta anevrizmal subaraknoid kanama tanısı ile izlenmektedir. Aşağıda bu
hastaya ait mevcut klinik veriler sunulmaktadır:

[HASTA VERİLERİ]

Bu verilere dayanarak hastada gecikmiş serebral iskemi (DCI) gelişme riskini
değerlendir.

KURALLAR:
- Yalnızca verilen verileri kullan. Varsayım yapma, ek bilgi üretme.
- Eksik veri için varsayım yapma veya değer uydurma; mevcut verilere göre
değerlendir.
- İstenen kategori ve sayıları net olarak ver; belirsiz ifadelerle yanıt verme.
- "Bu tıbbi tavsiye değildir" veya "bir hekime danışın" gibi sorumluluk reddi
cümleleri yazma.
- Tedavi önerisi, kaynak veya istenen format dışında hiçbir metin yazma.

YANIT FORMATI:
1. DCI gelişme risk kategorisi: Düşük / Orta / Yüksek
2. Tahmini DCI olasılığı (% olarak tek tam sayı, 0-100):
3. Bu DCI risk tahminine olan güven düzeyi (% olarak tek tam sayı, 0-100):
4. Klinik değerlendirme alanlarının DCI risk tahminine katkısı (her alan için
tek seçim):
   (1) Nörolojik şiddet: Destekler / Belirgin katkısı yok / Tahminle uyumsuz
   (2) Radyolojik yük: Destekler / Belirgin katkısı yok / Tahminle uyumsuz
   (3) İnflamatuvar yanıt: Destekler / Belirgin katkısı yok / Tahminle uyumsuz
   (4) Hemodinamik instabilite: Destekler / Belirgin katkısı yok / Tahminle
uyumsuz
   (5) Metabolik bozukluk: Destekler / Belirgin katkısı yok / Tahminle uyumsuz
```

A.2 Data field schema for the [HASTA VERİLERİ] field (field names and units; values left blank)

```
DEMOGRAFİ VE ÖYKÜ:
- Yaş: ___
- Cinsiyet: Erkek / Kadın
```

- Hipertansiyon: Var / Yok
- Diyabet: Var / Yok
- Koroner arter hastalığı: Var / Yok
- Diğer komorbidite: Var / Yok

ANEVRİZMA VE GİRİŞİM:
- Anevrizma lokalizasyonu: ___ (Anterior / Posterior dolaşım)
- Anevrizma sayısı: Tek / Çoklu
- Anevrizmanın güvene alınmasına kadar geçen süre (gün): ___
- Tedavi yöntemi: ___
- Antitrombotik kullanımı: Var / Yok (___)
- Nimodipin: Var / Yok
- Eksternal ventriküler drenaj (EVD): Var / Yok

BAŞVURU (BASELINE) DEĞERLENDİRMESİ:
- WFNS skoru: ___
- Modifiye Fisher skoru: ___
- SEBES skoru: ___
- İntraventriküler kanama (IVH): Var / Yok
- Hidrosefali: Var / Yok
- İntraparenkimal hematom (IPH): Var / Yok
- Midline shift: Var / Yok
- Başvuru GKS: ___
- Pupil: ___

GÜN [n] VERİLERİ:
- GKS: ___
- Sedasyon: Var / Yok
- Mekanik ventilasyon: Var / Yok
- Vazopressör: Var / Yok
- Enfeksiyon: Var / Yok
- Lökosit (WBC, $10^3/mm^3$): ___
- Nötrofil ($10^3/mm^3$): ___
- Lenfosit ($10^3/mm^3$): ___
- Nötrofil/Lenfosit oranı (NLR): ___
- C-reaktif protein (CRP, mg/L): ___
- Kreatinin (mg/dL): ___
- Sodyum (mmol/L): ___
- Glukoz (mg/dL): ___
- Laktat (mmol/L): ___

B.1 English prompt — standardized template (translation)

Evaluate this case as an intensive care specialist experienced in aneurysmal subarachnoid hemorrhage (aSAH).

This patient is being followed with a diagnosis of aneurysmal subarachnoid hemorrhage. The clinical data currently available for this patient are presented below:

[PATIENT DATA]

Based on these data, assess the risk of developing delayed cerebral ischemia (DCI) in this patient.

RULES:
- Use only the provided data. Do not make assumptions or generate additional information.

- Do not make assumptions or fabricate values for missing data; assess the patient based on the available data.
- Provide the requested categories and numbers clearly; do not respond with ambiguous wording.
- Do not write disclaimer sentences such as "this is not medical advice" or "consult a physician."
- Do not write any treatment recommendation, reference, or any text outside the requested format.

RESPONSE FORMAT:
1. DCI risk category: Low / Moderate / High
2. Estimated DCI probability (single integer percentage, 0-100):
3. Confidence in this DCI risk prediction (single integer percentage, 0-100):
4. Contribution of clinical evaluation domains to the DCI risk prediction (one selection per domain):
   (1) Neurological severity: Supports / No notable contribution / Inconsistent with the prediction
   (2) Radiological burden: Supports / No notable contribution / Inconsistent with the prediction
   (3) Inflammatory response: Supports / No notable contribution / Inconsistent with the prediction
   (4) Hemodynamic instability: Supports / No notable contribution / Inconsistent with the prediction
   (5) Metabolic derangement: Supports / No notable contribution / Inconsistent with the prediction

B.2 Data field schema for the [PATIENT DATA] field (field names and units; values left blank)

DEMOGRAPHICS AND HISTORY:
- Age: ___
- Sex: Male / Female
- Hypertension: Yes / No
- Diabetes: Yes / No
- Coronary artery disease: Yes / No
- Other comorbidity: Yes / No

ANEURYSM AND PROCEDURE:
- Aneurysm location: ___ (anterior / posterior circulation)
- Number of aneurysms: Single / Multiple
- Time to aneurysm securing (days): ___
- Treatment method: ___
- Antithrombotic use: Yes / No (___)
- Nimodipine: Yes / No
- External ventricular drain (EVD): Yes / No

ADMISSION (BASELINE) ASSESSMENT:
- WFNS grade: ___
- Modified Fisher scale: ___
- SEBES score: ___
- Intraventricular hemorrhage (IVH): Yes / No
- Hydrocephalus: Yes / No
- Intraparenchymal hematoma (IPH): Yes / No
- Midline shift: Yes / No
- Admission GCS: ___
- Pupils: ___

DAY [n] DATA:
- GCS: ___

- Sedation: Yes / No
- Mechanical ventilation: Yes / No
- Vasopressor: Yes / No
- Infection: Yes / No
- Leukocytes (WBC, $\times 10^3/mm^3$): ___
- Neutrophils ($\times 10^3/mm^3$): ___
- Lymphocytes ($\times 10^3/mm^3$): ___
- Neutrophil-to-lymphocyte ratio (NLR): ___
- C-reactive protein (CRP, mg/L): ___
- Creatinine (mg/dL): ___
- Sodium (mmol/L): ___
- Glucose (mg/dL): ___
- Lactate (mmol/L): ___
