# Supplementary material for: Phase-Based Dynamic Prediction of Delayed Cerebral Ischemia After Aneurysmal Subarachnoid Hemorrhage: Comparison of a Large Language Model with Intensive Care Specialists
Source: Medicina (Kaunas). 2026 Sep 2;62(9):1680. doi: 10.3390/medicina62091680 (PMC13609773; doi:10.3390/medicina62091680)
Supplement: Supplementary file 1 [file medicina-62-01680-s001.zip › Supplementary_File_S3_Adjudication_checklist.pdf]

Supplementary File S3

Structured adjudication checklist for delayed cerebral ischemia (DCI)

Phase-Based Dynamic Prediction of Delayed Cerebral Ischemia After Aneurysmal Subarachnoid Hemorrhage: Comparison of a Large Language Model With Intensive Care Specialists

The DCI outcome was adjudicated using the following structured checklist, which operationalizes the 2010 consensus definition of Vergouwen et al. Each case was reviewed independently by the three-member blinded adjudication panel; in the event of unanimous agreement the result was accepted directly, whereas disagreements were resolved by majority decision (2 of 3). The panel members were independent of the clinicians who provided the risk predictions. The Turkish original (as used) and its English translation are provided.

A. Turkish original (as used)

ADIM 1 – Zaman aralığı: Değerlendirme kanamadan sonraki 3–14 gün içerisinde mi? Bu aralık dışındaysa DCI dışı olarak sınıflandırılır.

ADIM 2 – Klinik kötüleşme: En az 1 saat süren yeni gelişen fokal nörolojik defisit veya GKS'de ≥2 puan düşüş var mı? Klinik kötüleşme yoksa görüntüleme bulguları ile birlikte değerlendirilir.

ADIM 3 – Alternatif nedenler: Rebleeding, hidrosefali, nöbet, metabolik bozukluk, enfeksiyon/sepsis veya hemodinamik instabilite klinik kötüleşmeyi açıklıyor mu? Açıklıyorsa DCI dışı olarak sınıflandırılır.

ADIM 4 – Görüntüleme: Takip görüntülemede yeni gelişen, anevrizmanın güvenceye alınması işlemine veya başka bir nedene bağlı olmayan serebral enfarkt var mı?

ADIM 5 – Nihai sınıflandırma: DCI / DCI dışı / Belirsiz.

B. English translation

STEP 1 – Time window: Is the assessment within days 3–14 after the hemorrhage? If outside this window, the case is classified as non-DCI.

STEP 2 – Clinical deterioration: Is there a new focal neurological deficit or a ≥2-point drop in the GCS lasting at least 1 hour? If there is no clinical deterioration, the case is evaluated together with the imaging findings.

STEP 3 – Alternative causes: Do rebleeding, hydrocephalus, seizures, metabolic derangement, infection/sepsis, or hemodynamic instability explain the clinical deterioration? If so, the case is classified as non-DCI.

STEP 4 – Imaging: Is there a new cerebral infarction on follow-up imaging that is not attributable to the aneurysm-securing procedure or another identifiable cause?

STEP 5 – Final classification: DCI / Non-DCI / Indeterminate.
