# Supplementary material for: Phase-Based Dynamic Prediction of Delayed Cerebral Ischemia After Aneurysmal Subarachnoid Hemorrhage: Comparison of a Large Language Model with Intensive Care Specialists
Source: Medicina (Kaunas). 2026 Sep 2;62(9):1680. doi: 10.3390/medicina62091680 (PMC13609773; doi:10.3390/medicina62091680)
Supplement: Supplementary file 1 [file medicina-62-01680-s001.zip › Supplementary_File_S4_TRIPOD-LLM_Checklist.pdf]

Supplementary File S4

TRIPOD-LLM reporting checklist

Phase-Based Dynamic Prediction of Delayed Cerebral Ischemia After Aneurysmal Subarachnoid Hemorrhage: Comparison of a Large Language Model With Intensive Care Specialists

Reporting follows TRIPOD-LLM (Gallifant J, et al. Nat Med 2025), an extension of TRIPOD+AI (Collins GS, et al. BMJ 2024). Study category: LLM evaluation; task: risk prediction/classification. Item texts are summarized in the authors' own words; an official numbered submission PDF can be generated from the interactive tool at tripod-llm.vercel.app. Section and line references refer to the submitted manuscript.

Section	No.	Item (summary)	Location in manuscript
Title	1	Identifies the study as an evaluation of an LLM; states the task (DCI prediction), target population (aSAH), and outcome	Title
Abstract	2	Structured abstract: design, task, data, model, performance, limitations	Abstract
Introduction	3a	Background: clinical context of DCI in aSAH and rationale for using an LLM	Introduction
	3b	Objectives / hypothesis (non-inferiority to specialists)	Introduction (final paragraph)
Materials and Methods	4a	Data source and study design (single-center, retrospective, phase-based)	Materials and Methods – Study design
	4b	Setting, locations, and periods	Materials and Methods – Study design
	5a	Participant eligibility criteria and selection	Materials and Methods – Patient selection; Figure 1
	5b	Data flow / inclusion–exclusion	Figure 1
	6	Outcome definition (DCI) and blinded adjudication panel; structured adjudication checklist	Materials and Methods – DCI definition and adjudication; Supplementary File S3
	7	Model inputs (clinical data) and information symmetry between assessors	Materials and Methods – Input/prompt; Assessors
	8	LLM details: model name/version (GPT-5.5 Thinking), access interface and dates, non-deterministic behavior	Materials and Methods – LLM/model
	9	Prompt strategy: standardized Turkish template, four-item output format, locked version	Materials and Methods – Prompt; Supplementary File S2 (template)
	10	Human oversight and comparator(s): two blinded intensive care specialists, expert average	Materials and Methods – Assessors
	11	Sample-size justification	Materials and Methods – Statistical analysis; Limitations
	12	Missing / indeterminate data handling	Materials and Methods – Statistical analysis; Table S3
	13	Analysis methods: discrimination (AUC, DeLong), non-inferiority (Δ = 0.10), calibration (Brier), DCA	Materials and Methods – Statistical analysis
	14	Task-specific performance and reproducibility (Fleiss κ, ICC; three runs)	Materials and Methods – Statistical analysis; Table 5
	15	Fairness / robustness and confidence-level assessment	Materials and Methods/Results; Tables S2, S4
Open science	16a	Ethics approval and informed consent / waiver	Institutional Review Board Statement; Informed Consent Statement (decision no. 9/2; 7 May 2026)

Section	No.	Item (summary)	Location in manuscript
	16b	Funding and competing interests	Funding; Conflicts of Interest
	16c	Protocol / preregistration status	Data Availability Statement
	16d	Availability of data, prompt, and code	Data Availability Statement; Supplementary File S2 (prompt)
Results	17a	Participant flow and characteristics	Results; Figure 1; Table 1
	17b	Discrimination results (LLM vs clinicians)	Results; Tables 2A–2B; Figure 2
	17c	Classification metrics and calibration	Results; Table 3; Figure S1
	17d	Non-inferiority and clinical utility (DCA)	Results; Table 2B; Figures S2–S3
	17e	Reproducibility, domain agreement, confidence, risk pattern, false reassurance	Results; Tables 4–5; Tables S1, S2, S4
Discussion	18	Interpretation (non-superiority; over-triage trade-off)	Discussion
	19a	Limitations (single-center, two clinicians, landmark, output granularity, measurement reliability)	Discussion – Limitations
	19b	Clinical applicability and human oversight (decision support, not autonomous)	Conclusions
