# Supplementary material for: Phase-Based Dynamic Prediction of Delayed Cerebral Ischemia After Aneurysmal Subarachnoid Hemorrhage: Comparison of a Large Language Model with Intensive Care Specialists
Source: Medicina (Kaunas). 2026 Sep 2;62(9):1680. doi: 10.3390/medicina62091680 (PMC13609773; doi:10.3390/medicina62091680)
Supplement: Supplementary file 1 [file medicina-62-01680-s001.zip › Supplementary_File_S5_STROBE_Checklist.pdf]

STROBE reporting checklist (cohort)

Phase-Based Dynamic Prediction of Delayed Cerebral Ischemia After Aneurysmal Subarachnoid Hemorrhage: Comparison of a Large Language Model With Intensive Care Specialists

Reporting follows the STROBE statement for observational (cohort) studies (von Elm E, et al.). Item texts are summarized in the authors' own words; full definitions are available at strobe-statement.org. Section and line references refer to the submitted manuscript.

Section	No.	Item (summary)	Location in manuscript
Title and abstract	1a	Study design indicated in the title/abstract with a common term	Title; Abstract
	1b	Balanced summary of what was done and found	Abstract
Introduction	2	Scientific background and rationale	Background
	3	Prespecified objectives / hypotheses (non-inferiority)	Background (final paragraph)
Methods	4	Study design early in the paper (single-center, retrospective, phase-based landmark)	Methods – Study design
	5	Setting, locations, and dates (admission, follow-up, data-collection periods)	Methods – Study design / Patient selection
	6a	Eligibility criteria, sources, and selection methods	Methods – Patient selection; Figure 1
	6b	Matching	Not applicable
	7	Variables: outcome (DCI), predictors (LLM/clinician risk assessment), diagnostic criteria	Methods – DCI definition; Assessors
	8	Data sources/measurement for each variable; comparability across groups (information symmetry)	Methods – Input/prompt; Adjudication
	9	Efforts to address potential sources of bias (blinding, phase concealment)	Methods – Adjudication / Design
	10	How the sample size was arrived at (no a priori power calculation; precision via 95% CIs)	Methods – Statistical analysis; Limitations
	11	Handling of quantitative variables and groupings (risk categories, probability ranges)	Methods – Input/prompt; Statistical analysis
	12a	All statistical methods (AUC, DeLong, non-inferiority Δ = 0.10, Brier, calibration, DCA)	Methods – Statistical analysis
	12b	Subgroups / interactions (exploratory: domain κ, confidence level)	Methods – Statistical analysis; Additional file 1: Tables S1–S2
	12c	Missing / indeterminate data handling (indeterminate cases; sensitivity analysis)	Methods – Statistical analysis; Additional file 1: Table S3
	12d	Follow-up / landmark approach	Methods – Study design; Limitations
	12e	Sensitivity analyses	Methods – Statistical analysis; Additional file 1: Table S3
Results	13a	Numbers of participants at each stage (assessed → included)	Results; Figure 1
	13b	Reasons for exclusion	Figure 1
	13c	Flow diagram	Figure 1
	14a	Participant characteristics and predictors	Results; Table 1
	14b	Missing data per variable	Methods – Statistical analysis (admission GCS in sedated patients treated as missing); Table 1 (Sedated/not evaluable, 23 [10.6%]); data complete for all other variables
	14c	Follow-up time (phase structure)	Methods – Study design
	15	Number of outcome events (DCI events)	Results; Table 1
	16a	Main results: estimates and 95% CIs (AUC, ΔAUC)	Results; Tables 2A–2B; Figure 2
	16b	Category boundaries (risk thresholds)	Methods/Results; Table 3

Section	No.	Item (summary)	Location in manuscript
	16c	Translation to absolute risk (calibration / DCA)	Results; Additional file 1: Figures S1, S3
	17	Other analyses (subgroup, sensitivity, reproducibility)	Results; Tables 4–5; Additional file 1: Tables S1–S4
Discussion	18	Key results summarized with reference to objectives	Discussion (opening)
	19	Limitations (single-center, two clinicians, landmark, output granularity)	Discussion – Limitations
	20	Cautious, holistic interpretation	Discussion
	21	Generalizability (case mix, clipping predominance)	Discussion – Limitations
Other information	22	Funding and role of the funder	Declarations
